# Insights Into Acute and Delayed Cisplatin-Induced Emesis From a Microelectrode Array, Radiotelemetry and Whole-Body Plethysmography Study of *Suncus murinus* (House Musk Shrew)

**DOI:** 10.3389/fphar.2021.746053

**Published:** 2021-12-03

**Authors:** Longlong Tu, Julia Y. H. Liu, Zengbing Lu, Dexuan Cui, Man P. Ngan, Peng Du, John A. Rudd

**Affiliations:** ^1^ School of Biomedical Sciences, The Chinese University of Hong Kong, Shatin, Hong Kong; ^2^ Auckland Bioengineering Institute, University of Auckland, Auckland, New Zealand.; ^3^ The Laboratory Animal Services Centre, The Chinese University of Hong Kong, Shatin, Hong Kong

**Keywords:** cisplatin, *Suncus murinus*, gastric myoelectric activity, respiratory activity, blood pressure, hypothermia, microelectrode assay

## Abstract

**Purpose:** Cancer patients receiving cisplatin therapy often experience side-effects such as nausea and emesis, but current anti-emetic regimens are suboptimal. Thus, to enable the development of efficacious anti-emetic treatments, the mechanisms of cisplatin-induced emesis must be determined. We therefore investigated these mechanisms in *Suncus murinus*, an insectivore that is capable of vomiting.

**Methods:** We used a microelectrode array system to examine the effect of cisplatin on the spatiotemporal properties of slow waves in stomach antrum, duodenum, ileum and colon tissues isolated from *S. murinus*. In addition, we used a multi-wire radiotelemetry system to record conscious animals’ gastric myoelectric activity, core body temperature, blood pressure (BP) and heart rate viability over 96-h periods. Furthermore, we used whole-body plethysmography to simultaneously monitor animals’ respiratory activity. At the end of *in vivo* experiments, the stomach antrum was collected and immunohistochemistry was performed to identify c-Kit and cluster of differentiation 45 (CD45)-positive cells.

**Results:** Our acute *in vitro* studies revealed that cisplatin (1–10 μM) treatment had acute region-dependent effects on pacemaking activity along the gastrointestinal tract, such that the stomach and colon responded oppositely to the duodenum and ileum. *S. murinus* treated with cisplatin for 90 min had a significantly lower dominant frequency (DF) in the ileum and a longer waveform period in the ileum and colon. Our 96-h recordings showed that cisplatin inhibited food and water intake and caused weight loss during the early and delayed phases. Moreover, cisplatin decreased the DF, increased the percentage power of bradygastria, and evoked a hypothermic response during the acute and delayed phases. Reductions in BP and respiratory rate were also observed. Finally, we demonstrated that treatment with cisplatin caused inflammation in the antrum of the stomach and reduced the density of the interstitial cells of Cajal (ICC).

**Conclusion:** These studies indicate that cisplatin treatment of *S. murinus* disrupted ICC networking and viability and also affected general homeostatic mechanisms of the cardiovascular system and gastrointestinal tract. The effect on the gastrointestinal tract appeared to be region-specific. Further investigations are required to comprehensively understand these mechanistic effects of cisplatin and their relationship to emesis.

## Introduction

The treatment of cancer with cisplatin-based therapy commonly causes side effects, such as severe nausea and emesis (retching and vomiting). These can be classified into “acute” and “delayed” phases: the acute phase comprises emetic episodes occurring during the first 24 h after beginning the treatment, and the delayed phase comprises those occurring from 24  to  120 h after beginning the treatment (Martin, 1996). Guidelines for the treatment of chemotherapy-induced acute and delayed emesis suggest the use of a combination of 5-HT_3_ antagonists (e.g., ondansetron or palonosetron) and NK_1_ receptor antagonists (e.g., aprepitant or netupitant), plus a glucocorticoid (e.g., dexamethasone) (Einhorn et al., 2017). However, the best combinations of anti-emetics are currently only effective in up to 80% of chemotherapy patients, and treatments for other causes of emesis (e.g., following surgery with anaesthesia and motion sickness) are also far from optimal (Aapro et al., 2013; Van den Brande et al., 2014). Whilst emesis is relatively easy to quantify, an assesment of nausea is more problematic and comparison across studies is difficult. Nevertheless, it is known that nausea remains a problem for some patients, even when their emesis is reduced (Andrews and Sanger, 2014). Therefore, further investigations are needed to fully elucidate the mechanisms by which cisplatin causes nausea and vomiting (Andrews and Sanger, 2014).

Cisplatin disrupts inter-digestive myoelectric activity and causes jejuno-gastric retrograde contraction during emesis in dogs (Chey et al., 1988), whereas nausea is commonly associated with myoelectrical dysrhythmias of the stomach in humans (Koch, 2014). In addition, our previous studies in ferrets revealed that cisplatin treatment reduced the dominant power (DP) in the stomach, concomitant with a decrease in the percentage of bradygastric power during the acute and delayed phases of the treatment, but had no effect on the dominant frequency (DF) or the percentage of normogastric or tachygastric power (Percie du Sert et al., 2009; Lu et al., 2017a). We further showed that cisplatin modified the multifractal magnitude of slow-wave structure, which was manifested as a reduction in slow-wave power and a shift to a simpler slow-wave shape (Lu et al., 2017a).

Clinical studies have shown that cisplatin may affect the autonomic nervous system and cause an increase in heart rate (HR) and a decrease in heart rate viability (HRV) (Morrow et al., 1999; Farmer et al., 2014). Similarly, we demonstrated that ferrets treated with cisplatin exhibited an (up to ∼50 bpm) increase in HR and a (∼25%) decrease in HRV during the acute phase of emesis (0–24 h) (Lu et al., 2017a). In contrast, there was a decrease in HR during the delayed phase of emesis (24–72 h) in these cisplatin-treated animals, but HRV was not altered (Lu et al., 2017a).

*Suncus murinus*, commonly known as the house musk shrew, is an insectivore that has been extensively used in research on the mechanisms of chemotherapy-induced nausea and vomiting (CINV) (Ueno et al., 1987; Matsuki et al., 1988). It would therefore be of interest to confirm the CINV findings previously obtained in this species, and to obtain additional information for anti-emetic research and on the ability of cisplatin to affect gastric slow-waves, as these waves, together with HRV and hypothermia, have been found to be associated with nausea and vomiting (Koch, 2014; Ngampramuan et al., 2014; Lu et al., 2016; Lu et al., 2017b). Accordingly, we simultaneously recorded the gastric myoelectric activity (GMA), core body temperature, BP and HRV of *S. murinus* using a multi-wire radiotelemetry system in experiments lasting 96 h (a 24-h baseline period, followed by a 72-h recording period subsequent to cisplatin administration). The dose of cisplatin (30 mg/kg, i.p.) we selected was based on previous studies where emesis was observed to occur over a 3-day period (Sam et al., 2003). Larger species including ferrets and cats would not survive such a high dose of cisplatin as toxicity, particularly to the kidney, would result in renal failure. (Rudd et al., 1994; Rudd et al., 2000; Eiseman et al., 2017). Based on doses calculated as mg/body surface area, the dose of 30 mg/kg (∼270–300 mg/m^2^) is acknowledged to be considerably higher (∼4 times) than doses of cisplatin causing emesis in man (Eiseman et al., 2017). The reason(s) underlying the species differences of mechanism of emetic sensitivity is unknown. Indeed, cisplatin at 30 mg/kg may be considered a borderline toxic dose in *Suncus murinus* and animals must be closely observed for humane endpoints and terminated at 3 days (Sam et al., 2003). It would not be possible to extend observation times past 3 days, nor give a repeated cycle of cisplatin in these animals. Indeed, some drugs, such as dexamethasone, paradoxically increase the toxicity of cisplatin which limits the translational value of the studies relevant to anti-emetic discovery (Sam et al., 2003). We also simultaneously recorded the respiratory function of *S. murinus* using whole-body plethysmography, which has previously been found to be altered during emesis in this species (Horn et al., 2016; Tu et al., 2017a). At the end of the experiments, the shrews were humanely sacrificed, and the antrum region of the stomach was isolated and processed to determine pathological changes, and immunohistochemistically stained to detect the presence of c-kit (a marker of ICC) and CD45 (a marker of inflammation). In addition, the effects of cisplatin on the spatiotemporal properties of slow-waves in isolated stomach antrum, duodenum, ileum and colon tissues were investigated using an MEA system (Liu et al., 2019). Acute effects (within minutes) of cisplatin on gastrointestinal tissue was investigated, but an *ex vivo* sampling time of 90 min from animals treated with cisplatin was also used, to correspond to a timepoint where the most intense vagally-mediated emetic response in this species is observed (Sam et al., 2003). We expected that these studies would yield new information on the mechanisms of emesis control related to respiratory activity, homeostasis and the gastrointestinal tract.

## Methods

### Animals

Adult male *S. murinus* (55–75 g) were obtained from the Chinese University of Hong Kong and housed in a temperature-controlled room (24 ± 1°C). Artificial lighting was provided between 06:00 and 18:00 h, and the relative humidity was maintained at 50 ± 5%. Water and dry-pelleted cat chow (Feline Diet 5003, PMI Feeds, St. Louis, United States) were given *ad libitum*, unless otherwise stated. All experiments were conducted under licence from the Government of the Hong Kong SAR and the Animal Experimentation Ethics Committee of The Chinese University of Hong Kong.

### Drug Formulation

Cisplatin (Merck, St. Louis, United States) was dissolved in acidified saline (154 mM NaCl, adjusted to pH 4.0 with 0.1 N HCl) with a final dose of 30 mg/kg (i.p., 10 ml/kg). The selection of 30 mg/kg is able to induce acute and delayed emesis reliably in *S. murinus* based on our previous studies (Sam et al., 2003; Chan et al., 2014).

### MEA

Electrical recordings were performed using an MEA, as previously described (Liu et al., 2019). In brief, intact full-thickness tissue segments of the antrum, duodenum, ileum and colon were isolated from the adult male shrews (55–75 g) and incubated in Kreb’s medium (in mM: NaCl, 115; KCl, 4.7; KH_2_PO_4_, 1.2; MgSO_4_.7H_2_O, 1.2; CaCl_2_.2H_2_O, 2.5; glucose, 10; NaHCO_3_, 25; sparged with 95%/5% oxygen/carbon dioxide. Tissue contents were washed out with Kreb’s medium via small openings at the fundus and at the pyloric sphincter of the stomach, or at the end of intestinal segments. Nifedipine (1 μM, Merck) was used to reduce smooth-muscle movements. An antrum segment was aligned facing the electrodes of an MEA60 chip (Ayanda Boisystems S.A.), or a 1-cm length of an intestinal segment was aligned with the lumen sitting horizontally across the MEA60 electrode recording area. The MEA60 chip consists of sixty 3-D tip-shaped microelectrodes of 30 µm diameter and 200 µm inter-electrode distance. The signals were recorded, digitised and amplified using an MEA1060 1200x (Multichannel Systems, Germany). The internal filter was customized to 0.05–3,000 Hz to fit the purpose of slow wave recordings. The temperature was maintained at 37.0°C. Pacemaker potentials were recorded at a sampling frequency of 1,000 Hz. This sampling frequency was considered enough to catch the pacemaker frequency at <1 Hz where aliasing artefact did not significantly alter the final signal shape. Raw data was directly recorded and saved without applying further filters using MC_Rack V4.6.2 (Multichannel Systems, Germany). The isolated GI tissues were tested separately using a randomized protocol to either 1 μM or 10 μM of cisplatin after 5 min baseline recordings, followed by another 7 min of post-drug administration recordings. No tissue was used more than once.

In other experiments, 20 adult male shrews (65–90 g) were treated with cisplatin (i.p., 30 mg/kg, *n* = 10) or saline vehicle (*n* = 10), and 90-min later were sacrificed by carbon dioxide asphyxiation. Tissues were collected and treated using a similar approach to that described above, and the baseline slow-wave signals of the tissues were then recorded for 5 min.

### Implantation of Radiotelemetry Transmitters

The surgical procedures were as described in our previous studies (Percie du Sert et al., 2010). In brief, animals were fasted overnight, injected with buprenorphine (Temgesic; 0.05 mg/kg, subcutaneous [s.c.]; Schering Plough, Welwyn Garden City, United Kingdom), anaesthetised with ketamine (20 mg/kg, intramuscular [i.m.]; Alfasan, Holland) and xylazine (3 mg/kg, i.m.; Alfasan, Holland), and maintained under anaesthesia with 3% isoflurane (Halocarbon Products Corporation, United States) in a 3:1 ratio of O_2_ to N_2_O using an anaesthetic machine (Narkomed 2C, Drager, United States). Following a midline abdominal incision, the distal stomach was exposed. Two biopotential wires of an HD-X11 transmitter (Data Sciences, Inc., United States) were inserted into the serosal wall of the antrum, and its catheter was inserted into the cervical artery up to a length of approximately 1.8 cm. A 1- × 1-cm piece of sterile gauze was placed over the catheter’s entry-point and fixed with a drop of tissue glue. The body of the transmitter was placed subcutaneously on the dorsal aspect of the animal. The abdominal cavity was closed using a continuous suture for the muscle layer and a discontinuous suture for the skin, and the initial incision was sprayed with a permeable spray dressing (Opsite, Smith and Nephew, United Kingdom). After surgery, all animals were administered marbofloxacin (Marbocyl, 2 mg/kg, s.c.) once per day for 3 days, and buprenorphine (0.05 mg/kg, s.c.) 12 h after the first dose. The animals were allowed 7 days to recover from these surgical procedures.

### Experimental Protocol

At approximately 9:00 am, the shrews were placed into a transparent Perspex whole-body plethysmography chamber (diameter, 19.1 cm; height, 14 cm; volume, 4,014.83 cm^3^; Data Sciences, Inc., United States) in which the airflow at 2.5 L/min was provided by a bias flow generator (Data Sciences, Inc., United States) and food and water were available *ad libitum*. After a 24-h period of baseline recordings, the animals were treated with cisplatin (30 mg/kg, i.p.) and then subjected to a 72-h period of recordings. Telemetric and respiratory data were acquired throughout the entire recording period. Emesis data were recorded for 72 h after cisplatin treatment via a video system (Panasonic WV-PC-240, China), and body weight and food and water intake were measured at 24-h intervals. All animals were terminated at 72 h by CO_2_ asphyxiation.

### Histological Studies

After the animals were sacrificed by CO_2_ asphyxiation, the antrum region of the stomach was collected and fixed in 4% paraformaldehyde, dehydrated and embedded in paraffin, and then divided into 6-µm sections. These sections were stained with haematoxylin and eosin (H&E), and then morphologically examined under a microscope (Carl Zeiss Axiophot 2 Upright Microscope, Carl Zeiss Inc., Thornwood, United States). Microscopy images were captured using a digital camera (Q-Imaging digital Camera).

For immunohistochemistry staining, the paraffin-embedded sections were deparaffinised, rehydrated, and then placed in 0.3% H_2_O_2_ for 10 min to block endogenous peroxidase activity. The sections were then washed with three successive volumes of phosphate-buffered saline (PBS). Antigen retrieval was conducted in a citrate buffer with a PH value of 6.0 at 95°C for at least 20 min (PT Module™, ThermoFisher, United States). Non-specific binding sites were blocked with 5% bovine serum albumin solution, and then CD45 and c-kit antibodies were added at a working concentration of 0.25 μg/ml (#13917, Cell Signaling Technology, United States) and 1.28 μg/ml (#3074, Cell Signaling Technology, United States), respectively, and the resulting mixtures left overnight at 4°C. The slides were then removed from their respective solutions, washed with PBS for 3 times, and then incubated with a secondary antibody for 2 h at room temperature. Next, the slides were developed with a 3,3′-Diaminobenzidine Substrate Kit (SK-4100, Vector Laboratories, California, United States) for 5 min, and then counterstained with haematoxylin. Stained slides were examined under a microscope, and the number of positive cells in each captured image were counted using ImageJ 1.51 (National Institutes of Health, Bethesda, MD, United States).

### Data Acquisition and Analysis

#### MEA Analysis

Raw data were recorded using MC_Rack, V4.6.2 (Multichannel Systems, Germany) and imported into Spike 2^®^ (Version 8.1, Cambridge Electronic Design, United Kingdom) for temporal analysis and into MATLAB (2016a, MathWorks) for spatial-temporal analysis, as previously described (Liu et al., 2019). In brief, a second-order Butterworth lowpass IIR filter at 100 Hz was applied to the raw data, which was then down-sampled to 100 Hz. A bandpass filter 0.05–0.5 Hz was furthered applied to down-sampled data. The DF was defined as the frequency with the highest power following a fast Fourier transform (FFT) -based (1,024-sample Hann window) spectrum analysis. The power spectrum was divided into brady-rhythm range frequencies [2 cycles per minute (cpm) to (DF − 1) cpm], normal-rhythm range frequencies [(DF ± 1) cpm], tachy-rhythm range frequencies [(DF + 1) cpm to 40 cpm] and out-of-range frequencies [(<2 cpm) and (>40 cpm)]. The average period of waveforms was derived by averaging the periods of all waveforms (or peak events) that were identified. The propagating velocity was defined by the movement of the same peak event horizontally/vertically across the MEA, as previously described (Liu et al., 2019).

#### Radiotelemetry

GMA data were initially analysed using Spike2 (Version 8.1, Cambridge Electronic Design, United Kingdom), using methods previously developed by our laboratory (Percie du Sert et al., 2010; Tu et al., 2017a). In brief, gastric slow-waves were recorded at a sampling frequency of 1,000 Hz, and were then filtered in several steps to a 0.03–0.5 Hz (2–30 cpm) window, and subsequently down-sampled to 10.24 Hz to remove cardiac and respiratory signals and low-frequency artefacts (such as movement). FFTs (2,048-sample Hann window) were computed for successive 10-min sections of the data. The following parameters were used to characterise the GMA data: DF – the frequency bin with the highest power in the 2–24 cpm range; DP – the highest power in the 2–24 cpm range; and the repartition of power in bradygastric (2–DF-2 cpm), normal (DF-2–DF + 2 cpm) and tachygastric (DF + 2–24 cpm) ranges (i.e. bradygastria, normogastria and tachygastria). All data collected by radiotelemetry, including body temperature data, were averages of data recorded every 10 min.

We also used advanced analytical techniques to examine the structure of slow waves. Thus, multifractal detrended fluctuation analysis (MFDFA) was used to obtain singularity spectra (α) (Kantelhardt et al., 2002), as a multifractal spectrum identifies the deviation in fractal structure over time by comparing large and small fluctuations (Ihlen, 2012). Generally, the multifractal spectra [plot *f(*α*) vs.* α] of signals with multifractal organisation have a concave-downward curvature. The width (α = α *max*–α *min*) of singularity spectra can be used to characterise the spectra, as a measure of the complexity of the multifractal process. We used Spike2 with custom scripts to perform MFDFA. The code for MFDFA in the Spike2 software was provided in the Supplementary Material.

#### Respiration

Compensated whole-body plethysmography (500-05RevA, Data Sciences, Inc., United States) has been used in studies of respiratory functions implicated in emesis in *S. murinus* (Tu et al., 2017a). In brief, the system we used consisted of two transparent chambers, each equipped with a Validyne pressure transducer (600–900 mmHg), a temperature sensor (0–100°C), and a humidity sensor (0–100%). All channel signals from the two chambers were collected using an ACQ7700 Carrier and a UniversalXE Signal Conditioner connected to a Micro 1401 data acquisition unit (Cambridge Electronic Design, United Kingdom). Signals were thereafter acquired and analysed using Spike2. As body temperature is required for calculating respiratory tidal volume, the real-time body temperature data from the telemetry transmitters were calculated using offline processing software [Microsoft Excel 2010 (v14.0, United States )].

#### Analysis of Emetic Data Using Burst Analysis

Burst analysis of emetic data was as previously performed in studies of emesis in *S. murinus* (Tu et al., 2017a). In brief, six parameters were defined to enable automated burst analysis using Spike2: the events per episode, the mean inter-event duration, the mean retch/vomit frequency, episode duration, the interval between episodes (the time between the end of the most recent episode to the onset of the next episode) and the cycles between episodes (the time between the onset of the most recent episode to the onset of the next episode).

### Statistical Analyses

All statistical analyses were performed using GraphPad Prism version 7 (GraphPad Prism version 7.0, Inc. California, United States). Differences in food and water consumption, body weight, mean arterial BP, HR, HRV, core body temperature, GMA and ∆α between baseline and post-cisplatin injection in saline groups were assessed using a one-way analysis of variance (ANOVA), followed by Bonferroni’s multiple comparison tests, or by paired Student’s *t*-tests, as appropriate. Differences in the MEA data were assessed using paired *t*-tests to compare the baseline and the post-drug recordings. All data from the *in vivo* studies are expressed as means ± standard errors of the means (SEMs), while data from the MEA analysis are expressed as means ± standard deviations (SDs). Differences were considered statistically significant at *p* < 0.05.

## Results

### Effects of Acute Cisplatin Treatment on Slow Waves in the Gastrointestinal Tract of *S. murinus*

#### Acute *in vitro* Effects of Acute Cisplatin Treatment on the DF

Treatment with 10 μM cisplatin significantly increased the DF in the stomach (from 5.8 ± 6–6.6 ± 1.0 cpm; *p* < 0.01, *n* = 14) and in the colon (from 24.6 ± 0.9–26.8 ± 1.0 cpm; *p* < 0.001, *n* = 7), but treatment with 1 μM cisplatin did not significantly affect the DF in the stomach (*p* > 0.05, *n* = 13) or colon (*p* > 0.05, *n* = 6) (Tables 1, 2). Treatment with 10 μM cisplatin had a different effect on the duodenum, as it significantly decreased the DF (from 26.2 ± 1.5 to 24.6 ± 1.4 cpm; *p* < 0.05, *n* = 8), but treatment with 1 μM cisplatin had no effect (*p* > 0.05, *n* = 6). Cisplatin (1–10 μM) had no effect on the DF in the ileum (1 μM, *p* > 0.05, *n* = 6; 10 μM, *p* > 0.05, *n* = 9) (Tables 1, 2).

**TABLE 1:** Effect of cisplatin (1 μM) on the dominant frequency (DF), average period and propagating velocity, compared to baseline.

	DF (cpm)		Average period (s)		Propagating velocity (mm s^−1^)		Number of repeats (*n*)
	Baseline	Cisplatin (1 μM)	Baseline	Cisplatin (1 μM)	Baseline	Cisplatin (1 μM)	
Stomach	7.6 ± 1.6	7.6 ± 2.3	4.9 ± 0.7	4.7 ± 0.7	19.9 ± 17.7	9.4 ± 9.0	13
Duodenum	26.6 ± 2.2	25.6 ± 1.3	2.3 ± 0.2	2.0 ± 0.4	35.0 ± 27.3	39.4 ± 27.6	6
Ileum	25.9 ± 2.9	21.9 ± 5.9	2.4 ± 0.2	2.6 ± 0.2^*^	10.0 ± 11.2	9.3 ± 9.3	6
Colon	25.2 ± 1.2	24.9 ± 1.3	2.4 ± 0.2	2.7 ± 0.2^*^	3.1 ± 0.3	3.9 ± 0.7	6

Data are mean values ± standard deviations. Significant differences relative to the baseline are indicated as * *p* < 0.05, ** *p* < 0.01 or *p* < 0.001 (paired *t*-tests).

**TABLE 2:** Effect of cisplatin (10 μM) on the dominant frequency (DF), average period and propagating velocity, compared to baseline.

	DF (cpm)		Average period (s)		Propagating velocity (mm s^−1^)		Number of repeats (*n*)
	Baseline	Cisplatin (10 μM)	Baseline	Cisplatin (10 μM)	Baseline	Cisplatin (10 μM)	
Stomach	5.8 ± 0.6	6.6 ± 1.0^**^	4.6 ± 0.4	4.4 ± 0.4	3.9 ± 5.2	4.2 ± 3.4	14
Duodenum	26.2 ± 1.5	24.6 ± 1.4^*^	2.3 ± 0.1	2.5 ± 0.2^*^	36.1 ± 28.0	75.4 ± 53.9^*^	8
Ileum	24.6 ± 1.6	22.4 ± 4.2	2.4 ± 0.1	2.8 ± 0.3^**^	29.9 ± 19.1	24.4 ± 34.7	9
Colon	24.6 ± 0.9	26.8 ± 1.0^***^	2.4 ± 0.1	2.4 ± 0.1	8.6 ± 4.4	5.3 ± 3.5^*^	7

Data are mean values ± standard deviations. Significant differences relative to the baseline are indicated as * *p* < 0.05, ** *p* < 0.01 or *p* < 0.001 (paired *t*-tests).

In relative terms, treatment with cisplatin (1–10 μM) increased the DF of pacemaker potentials in the antrum (1 μM: 62% increase, 15% no change, 23% decrease, *n* = 13; 10 μM: 79% increase, 0% no change, 21% decrease, *n* = 14) and colon (1 μM: 33% increase, 17% no change, 50% decrease, *n* = 7; 10 μM: 100% increase, 0% no change, 0% decrease, *n* = 6), but decreased the DF of pacemaker potentials in the duodenum (1 μM: 33% increase, 17% no change, 50% decrease, *n* = 6; 10 μM: 13% increase, 0% no change, 88% decrease, *n* = 8) and ileum (1 μM: 0% increase, 17% no change, 83% decrease, *n* = 6; 10 μM: 11% increase, 33% no change, 56% decrease, *n* = 9). Representative raw traces of pacemaker potentials at baseline and during cisplatin treatment were shown in Supplementary Figure S1.

#### Acute *In Vitro* Effects of Acute Cisplatin Treatment on Average Period of Waveforms

Cisplatin (1–10 μM) significantly increased the average period of waveforms in the ileum, from 2.4 ± 0.2–2.6 ± 0.2 s (1 μM, *p* < 0.05, *n* = 6), and from 2.4 ± 0.1–2.8 ± 0.3 s (10 μM, *p* < 0.01, *n* = 9) (Tables 1, 2). Cisplatin at 10 μM significantly increased the average period of waveforms in the duodenum from 2.3 ± 0.1–2.5 ± 0.2 s (*p* < 0.05, *n* = 8), but no significant differences were found after 1 μM treatment (*p* > 0.05, *n* = 6) (Tables 1, 2). Cisplatin at 1 μM significantly increased the average period of waveforms in the colon from 2.4 ± 0.2–2.7 ± 0.2 s (*p* < 0.05, *n* = 6), but no significant differences were found after 10 μM treatment (*p* > 0.05, *n* = 7) (Tables 1, 2). In the stomach, cisplatin (1–10 μM) did not change the average period of waveforms (1 μM, *p* > 0.05, *n* = 13; 10 μM, *p* > 0.05, *n* = 14) (Tables 1, 2).

#### Acute *In Vitro* Effects of Acute Cisplatin Treatment on Propagating Velocity

Cisplatin at 10 μM significantly increased the propagating velocity in the duodenum (from 36.1 ± 28.0–75.4 ± 53.9 mm s^−1^, *p* < 0.05, *n* = 8), but reduced the propagating velocity in the colon (from 8.6 ± 4.4–5.3 ± 3.5 mm s^−1^, *p* < 0.05, *n* = 7). However, no significant differences in the propagating velocity were found after 1 μM treatment in either the duodenum (*p* > 0.05, *n* = 6) or colon (*p* > 0.05, *n* = 6) (Tables 1, 2). In the stomach and ileum, cisplatin (1–10 μM) did not change the propagating velocity (stomach: 1 μM, *p* > 0.05, *n* = 13; 10 μM, *p* > 0.05, *n* = 14; ileum: 1 μM, *p* > 0.05, *n* = 6; 10 μM, *p* > 0.05, *n* = 9) (Tables 1, 2).

#### Acute *In Vitro* Effects of Acute Cisplatin Treatment on Frequency Distributions

**FIGURE 1:**
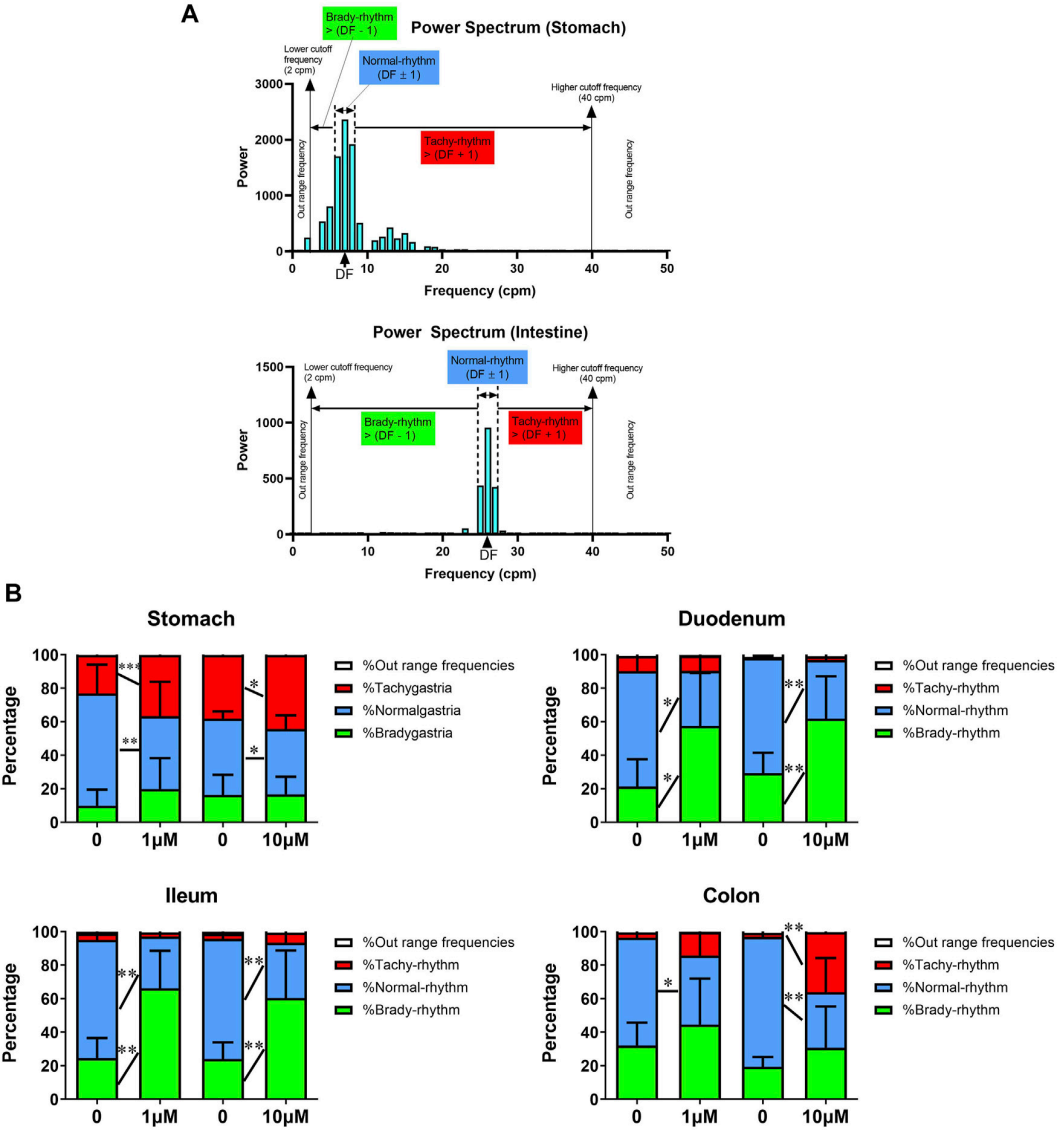
Acute *In vitro* effects of cisplatin on pacemaking potentials in the gastrointestinal tract of *Suncus murinus*. **(A)** A representative power spectrum of the **(upper)** stomach or **(lower)** intestinal segment in *S. murinus*. The power distribution was defined as brady-rhythm range frequencies [2 cpm to (dominant frequency (DF)) − 1)]; normal-rhythm range frequencies [(DF − 1) to (DF + 1)]; tachy-rhythm range frequencies [(DF + 1) to 40 cpm]; and out-of-range frequencies [(<2 cpm) and (>40 cpm)]. **(B)** Graphs showing the effect of cisplatin (1–10 μM) on the percentage of each range in the power spectrum in the stomach (1 μM: *n* = 13; 10 μM: *n* = 14), duodenum (1 μM: *n* = 6; 10 μM: *n* = 8), ileum (1 μM: *n* = 6; 10 μM: *n* = 9) and colon (1 μM: *n* = 7; 10 μM: *n* = 6). Data are mean values ± standard deviations. Significant differences relative to the baseline are indicated as ^*^ *p* < 0.05, ^**^ *p* < 0.01 or ^***^ *p* < 0.001 (paired *t*-tests).

Analysing the power spectrum of pacemaking potentials reveals the drug-induced changes in the pacing or rhythm of the gastrointestinal tract. The DF was defined by the basal recordings for each separate experiment and was approximately 6–8 cpm in the stomach and 21–27 cpm in the intestines (Figure 1A).

In the stomach, cisplatin (1–10 μM) did not change the percentage of brady-rhythm range frequencies, but significantly increased the percentage of tachy-rhythm range frequencies (from 38.1 ± 11.1% to 44.1 ± 12.0% at 1 μM, *p* < 0.001, *n* = 13 and from 23.0 ± 13.5% to 36.5 ± 12.1% at 10 μM, *p* < 0.05, *n* = 14). It also caused a significant decrease in the percentage of normal-rhythm range frequencies from 45.5–4.3 to 39.0 ± 8.1% at 1 μM (*p* < 0.01, *n* = 13) and from 67.0 ± 17.1%–43.6 ± 20.4% at 10 μM (*p* < 0.05, *n* = 14) (Figure 1B). In the duodenum, cisplatin (1–10 μM) did not change the percentage of tachy-rhythm range frequencies, but significantly increased the percentage of brady-rhythm range frequencies from 21.4 ± 16.3% to 57.5 ± 31.5% at 1 μM (*p* < 0.05, *n* = 6) and from 29.3 ± 12.1% to 61.8 ± 25.3% at 10 μM (*p* < 0.01, *n* = 8). It also caused a significant decrease in the percentage of normal-rhythm range frequencies (from 68.9 ± 18.2 to 32.9 ± 21.3% at 1 μM, *p* < 0.05, *n* = 6 and from 68.8 ± 12.0% to 35.0 ± 23.7% at 10 μM, *p* < 0.01, *n* = 8) (Figure 1B). In the ileum, cisplatin (1–10 μM) did not change the percentage of tachy-rhythm range frequencies, but significantly increased the percentage of brady-rhythm range frequencies (from 24.6 ± 11.9% to 66.2 ± 22.4% at 1 μM, *p* < 0.01, *n* = 6 and from 24.1 ± 9.9% to 60.4 ± 28.4% at 10 μM, *p* < 0.01, *n* = 9). It also caused a significant decrease in the percentage of normal-rhythm range frequencies (from 70.4 ± 12.5 to 30.8 ± 22.1% at 1 μM, *p* < 0.01, *n* = 6 and from 71.8 ± 9.5% to 33.1 ± 30.0% at 10 μM, *p* < 0.01, *n* = 9) (Figure 1B). In the colon, cisplatin at 1 μM significantly decreased the percentage of normal-rhythm range frequencies (from 64.3.4 ± 14.3 to 41.1 ± 26.1%, *p* < 0.05, *n* = 6), but did not change the percentage of tachy-rhythm and brady-rhythm range frequencies, whereas cisplatin at 10 μM significantly increased the percentage of tachy-rhythm range frequencies (from 2.6 ± 3.2 to 45.7 ± 23.2%, *p* < 0.01, *n* = 7) and also caused a significant decrease in the percentage of normal-rhythm range frequencies (from 76.2 ± 3.8 to 33.8 ± 23.3%, *p* < 0.01, n = 7) (Figure 1B).

#### Acute *In Vitro* Effects of Acute Cisplatin Treatment on Wave Propagation

**FIGURE 2:**
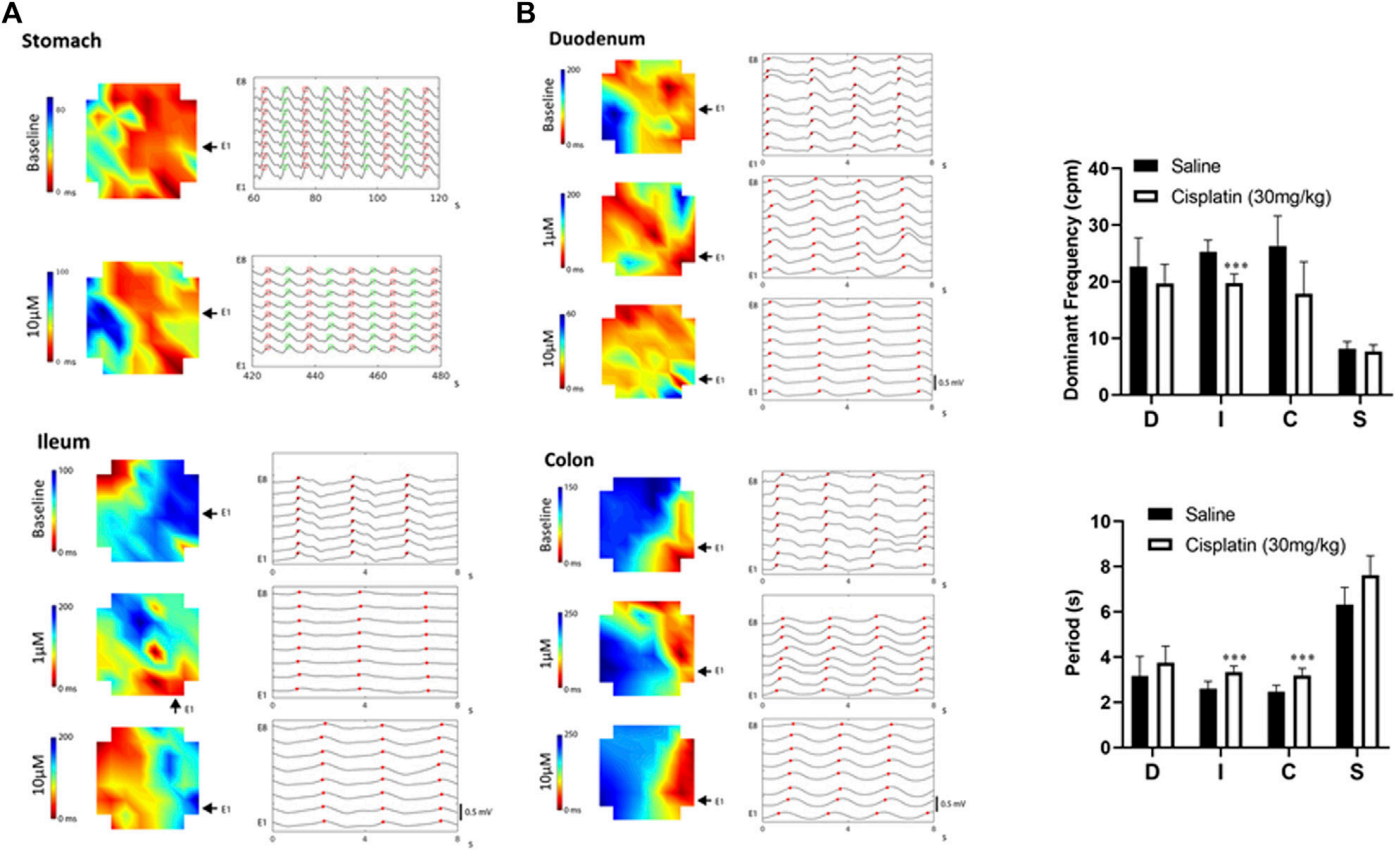
**(A)** Diagram showing representative activation maps indicating the spread of slow waves (red to blue) over time. Propagation became faster in the duodenum, but became slower in the colon or stomach following cisplatin (1–10 μM). The same peak events are labelled with red dots (plus green dots in stomach) and aligned vertically and separated by time across a horizontal/vertical array of eight electrodes. The raw traces of the stomach represent 60 s of data, while the raw traces of the duodenum, ileum and colon represent 8 s of data. **(B)** *In vivo* effects of cisplatin treatment (30 mg/kg, i.p.) at 90 min. Slow-wave signals were measured using microelectrode array techniques. Data are means ± standard deviations. Significant differences relative to the baseline are indicated as ^***^ *p* < 0.001 (unpaired *t*-tests). D: duodenum; I, ileum; C, colon; S, stomach.

Spatiotemporal analysis of the MEA data revealed that cisplatin (1–10 μM) increased the propagating velocity in the duodenum and ileum, but decreased the propagating velocity in the stomach and colon (Figure 2A). Thus, cisplatin (1–10 μM) decreased the time taken for a wavefront to move across the array of electrodes (red to blue) in the duodenum and ileum, but increased the time taken for a wavefront to move across this array in the stomach (10 μM) and colon (1–10 μM) (Figure 2A).

### Effect of Cisplatin Assessed *Ex Vivo* at 90 min

Compared with *S. murinus* treated with a vehicle control, those treated with cisplatin (30 mg/kg, intraperitoneal) for 90 min had a significantly lower DF in the ileum (vehicle: 25.3 ± 2.0 cpm vs. cisplatin: 19.7 ± 1.7 cpm; *p* < 0.001, *n* = 10), but treatment effects on the stomach (vehicle: 8.1 ± 1.3 cpm vs. cisplatin: 7.7 ± 1.2 cpm), duodenum (vehicle: 22.7 ± 5.0 cpm vs. cisplatin: 19.6 ± 3.5 cpm) and colon (vehicle: 26.3 ± 5.3 cpm vs. cisplatin: 17.9 ± 5.6 cpm) were not significant (*p* > 0.05, *n* = 10) (Figure 2B). Cisplatin also significantly increased the period of waveforms in the ileum (vehicle: 2.6 ± 0.3 s vs. cisplatin: 3.3 ± 0.3 s) and colon (vehicle: 2.5 ± 0.3 s vs. cisplatin: 3.2 ± 0.3 s) compared to a vehicle control (*p* < 0.001, *n* = 10), but treatment effects on the stomach (vehicle: 6.3 ± 0.8 s vs. cisplatin: 7.6 ± 0.8 s) and duodenum (vehicle: 3.2 ± 0.9 s vs. cisplatin: 3.8 ± 0.7 s) were not significant (*p* > 0.05, *n* = 10) (Figure 2B).

### *In Vivo* Effect of Cisplatin on Emesis, Body Weight, and Food and Water Intake

Cisplatin treatment (30 mg/kg, i.p.) induced emesis in all animals (*n* = 7) with a median latency of 45.1 min, while animals treated with vehicle (*n* = 8) did not exhibit emesis during the 72-h observation period. During the acute phase of emesis (0–24 h), cisplatin induced ∼13 episodes of emesis that comprised ∼88 retches and vomits. Moreover, ∼two and three episodes of emesis, comprising ∼17 and ∼25 retches and vomits, were observed during the 24–48-h and 48–72-h periods, respectively. The profile of cisplatin- and vehicle-induced retching + vomiting is shown in Supplementary Figure S2.

Cisplatin caused a significant reduction in body weight during the 48–72-h period after treatment (−24–0 h vs. 48–72 h: 69.1 vs. 59.2 g, *p* < 0.01) (Table 3). Compared with baseline measurements at −24–0 h (102.1 g/kg), a decrease in food intake was observed after treatment with cisplatin during the 0–24-h (48.7 g/kg, *p* < 0.001), 24–48-h (59.0 g/kg, *p* < 0.01) and 48–72-h (30.2 g/kg, *p* < 0.001) periods (Table 3). Additionally, in comparison with baseline measurements at −24–0 h (223.7 ml/kg), animals treated with cisplatin had a significantly lower water intake during the 0–24-h (96.0 ml/kg, *p* < 0.001), 24–48-h (126.3 g/kg, *p* < 0.001) and 48–72-h (105.0 ml/kg, *p* < 0.001) periods (Table 3). While vehicle treatment did not modify the animals’ body weight or eating or drinking behaviour (*p* > 0.05) (Table 3).

**TABLE 3:** Effect of vehicle or cisplatin (30 mg/kg, intraperitoneal) on the body weight and food and water intake of *Suncus murinus*.

	Body weight (g)		Food Intake (g·kg^−1^)		Water Intake (ml·kg^−1^)	
	Vehicle	Cisplatin	Vehicle	Cisplatin	Vehicle	Cisplatin
-24–0 h	70.1 ± 1.6	69.1 ± 1.7	95.2 ± 4.8	102.1 ± 9.5	199.6 ± 14.1	223.7 ± 3.3
0–24 h	69.9 ± 1.7	64.5 ± 1.9	94.9 ± 7.0	48.7 ± 10.0^***###^	203.2 ± 16.0	96.0 ± 7.7^***###^
24–48 h	69.9 ± 2.4	63.3 ± 1.7	92.1 ± 6.6	59.0 ± 7.8^*##^	180.5 ± 11.2	126.3 ± 17.4^###^
48–72 h	70.8 ± 2.2	59.2 ± 2.0^***##^	101.4 ± 4.6	30.2 ± 13.8^***###^	184.5 ± 16.2	105.0 ± 17.8^***###^

Data are means ± standard errors of the mean for 7 – 8 animals. Significant differences relative to the baseline (-24–0 h) for each group are shown as ^##^ *p* < 0.01 or ^###^ *p* < 0.001; Significant differences between cisplatin and vehicle groups are shown as ^*^ *p* < 0.05, ^**^ *p* < 0.01 or ^***^ *p* < 0.001 (two-way analysis of variance, followed by Bonferroni tests).

### *In Vivo* Effect of Cisplatin on GMA and Body Temperature

**FIGURE 3:**
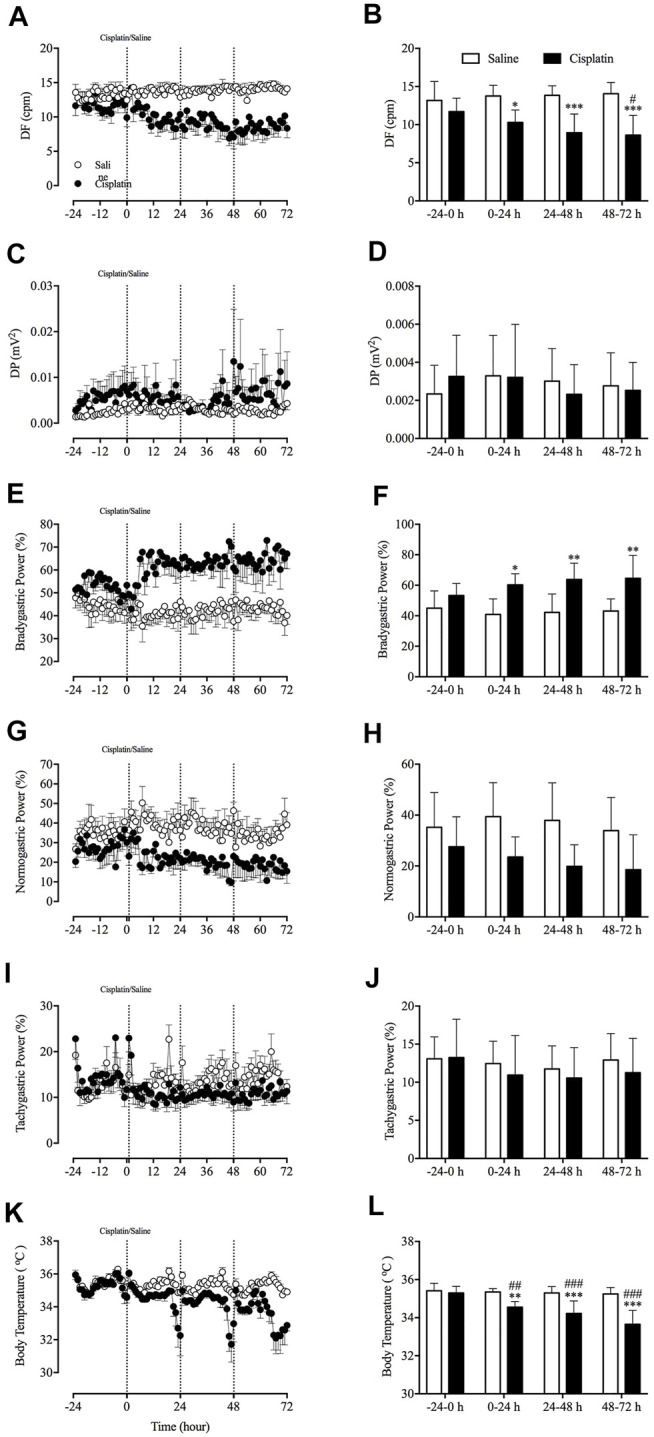
Effect of cisplatin (30 mg/kg, intraperitoneal) on gastric myoelectric activity in *Suncus murinus*. **(A)** The dominant frequency (DF); **(B)** the change in the DF averaged over 24 h; **(C)** the dominant power (DP); **(D)** the change in the DP averaged over 24 h; **(E)** bradygastria (%); **(F)** the change in bradygastria averaged over 24 h; **(G)** normogastria (%); **(H)** the change in normogastria averaged over 24 h; **(I)** tachygastria (%); **(J)** the change in tachygastria averaged over 24 h; (**K)** body temperature; and **(L)** the change in body temperature averaged over 24 h cpm = cycles per min. Data are means ± standard errors of the mean for *n* = 7 – 8 animals. Significant differences relative to baseline (-24–0 h) in the groups are shown as ^#^ *p* < 0.05, ^##^ *p* < 0.01, ^###^ *p* < 0.001 [two-way analysis of variance (ANOVA) followed by Bonferroni tests]. Significant differences between cisplatin and saline groups are shown as ^*^ *p* < 0.05, ^**^ *p* < 0.01 or ^***^ *p* < 0.001 (two-way ANOVA followed by Bonferroni tests).

**FIGURE 4:**
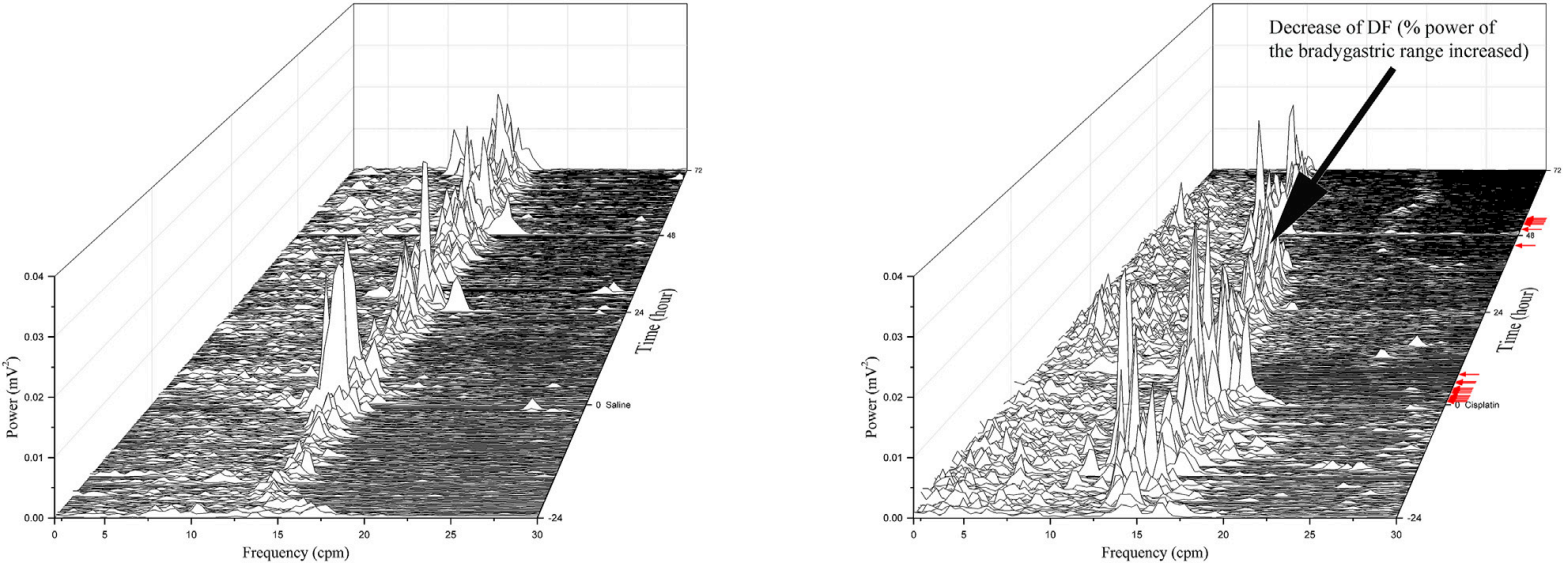
Running spectrum analysis of gastric myoelectric activity in vehicle- **(A)** and cisplatin-treated **(B)** *Suncus murinus*. This cisplatin-treated animal exhibited emesis after a latency of 37.3 min, and it had 12 and 7 episodes of emesis during the 0–24 h and 24–72 h periods, respectively. Note the decrease in the DF following cisplatin administration. Red arrows indicate where emetic events occurred after treatment with cisplatin.

Prior to randomisation to different treatment groups, the baseline GMA recordings (-24–0 h) revealed a DF of 12.6 ± 0.7 cpm concomitant with a DP of 2.6 × 10^-3^ ± 1.7 × 10^-3^ mV^2^, where 48.4 ± 3.0% of power was in the bradygastric range, 32.0 ± 3.7% of power was in the normogastric range, and 13.1 ± 1.1% of power was in the tachygastric range (pooled data, *n* = 15). Compared with animals treated with vehicle, those treated with cisplatin showed a significant reduction in the DF during the 0–24-h (13.76 vs. 10.30 cpm, *p* < 0.05), 24–48-h (13.85 vs. 8.94 cpm, *p* < 0.001) and 48–72 h (14.05 vs. 8.63 cpm, *p* < 0.001) periods (Figures 3A,B). Moreover, cisplatin caused a significant increase in the power of the bradygastric range during the 0–24-h (40.91 vs. 60.23%, *p* < 0.05), 24–48-h (42.17 vs. 63.78%, *p* < 0.001) and 48–72-h (43.15 vs. 64.56%, *p* < 0.001) periods (Figures 3E,F). In contrast, a non -significant reduction in the power in the normogastric range was observed in cisplatin-treated animals during the 0–24-h (39.41 vs. 23.53%, *p* = 0.14), 24–48-h (37.90 vs. 19.86%, *p* = 0.07) and 48–72-h (33.90 vs. 18.52%, *p* = 0.16) periods (Figures 3G,H). Neither vehicle nor cisplatin modified the DP or power in the tachygastric range during the 72-h observation period after treatment (Figures 3C,D, 3I,J). A representative running spectrum analysis (RSA) of the GMA of one saline- and one cisplatin-treated animal is shown in Figure 4.

The MFDFA analysis of GMA did not reveal significant differences between the width of the singularity strength Δα in animals treated with cisplatin or vehicle during the 72-h observation period (*p* > 0.05) (Supplementary Figure S3C). Representative multifractal spectrum graphs of a cisplatin-treated animal are shown in Supplementary Figures S3A,B.

Analysis of data around and during cisplatin-induced emetic episodes revealed a significant increase of DF before emetic events (7.95 vs. 12.08, *p* < 0.01) in comparison with baseline (Supplementary Figure S4A). Moreover, a 98% increase before emesis, and an 85% increase after emesis in the % power of tachygastria were observed (*p* < 0.05) (Supplementary Figure S4D), without affecting of the % power of bradygastria or normogastria (*p* > 0.05) when compared to baseline (Supplementary Figures S4B,C).

The radiotelemetric recording revealed a body temperature of 35.4 ± 0.1°C during the baseline (-24–0 h) period (pooled data, *n* = 15). Compared with animals treated with vehicle, those treated with cisplatin showed a significant decrease body temperature during the 0–24-h (35.35 vs. 34.55°C, *p* < 0.01), 24–48-h (35.30 vs. 34.21°C, *p* < 0.001) and 48–72-h (35.24 vs. 33.65°C, *p* < 0.001) post-treatment periods (Figures 3K,L). Animals treated with vehicle had a relatively stable body temperature throughout the entire experiment.

### *In Vivo* Effects of Cisplatin on Cardiovascular Homeostasis

**FIGURE 5:**
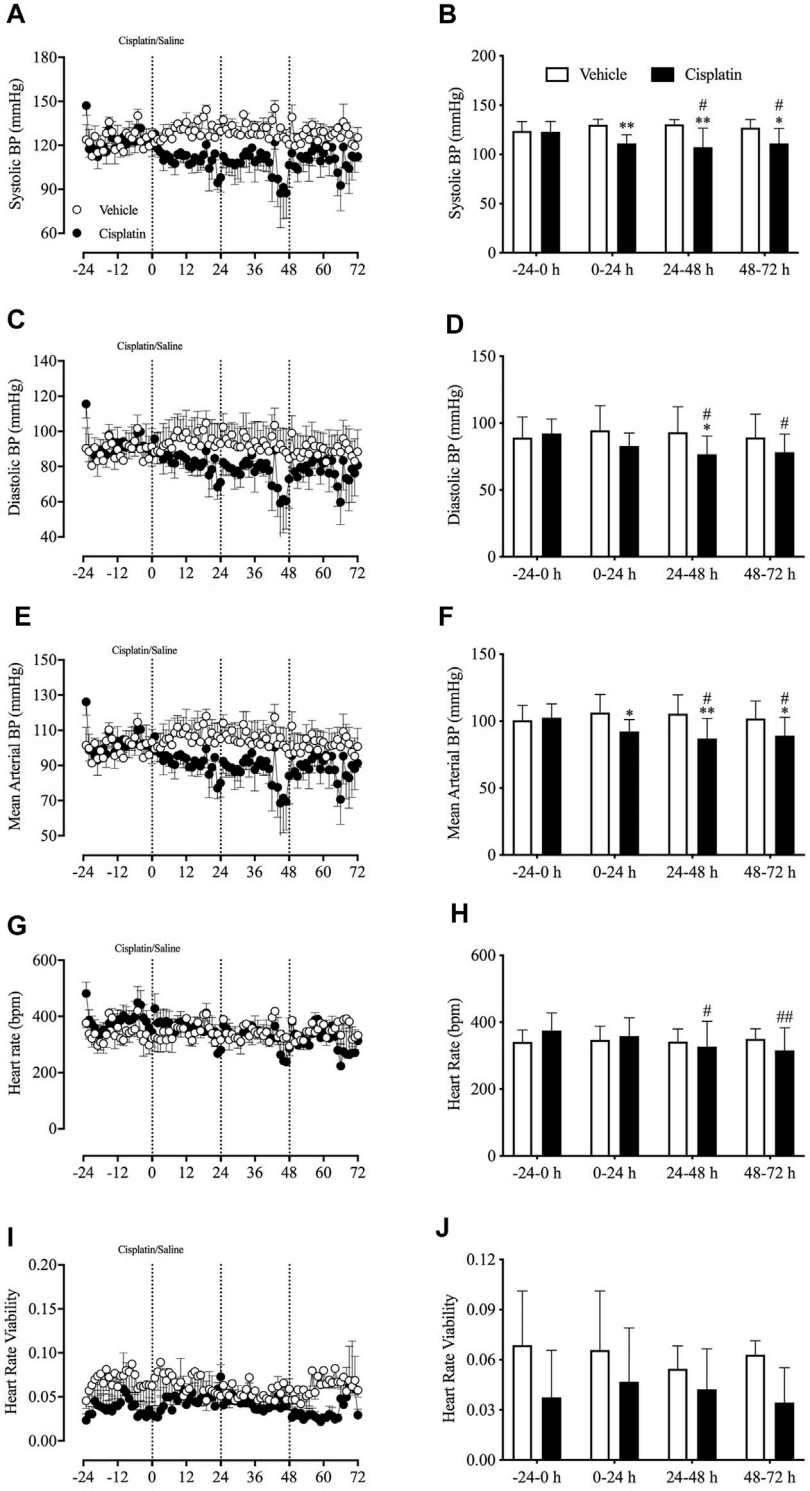
Effect of cisplatin (30 mg/kg, intraperitoneal) on cardiovascular homeostasis in *Suncus murinus*. **(A)** Systolic blood pressure (BP); **(B)** change in systolic BP averaged over 24 h; **(C)** diastolic BP; **(D)** change in diastolic BP averaged over 24 h; **(E)** mean arterial BP; **(F)** change in mean arterial BP averaged over 24 h; **(G)** heart rate (HR); **(H)** change in HR averaged over 24 h; **(I)** heart rate viability (HRV); **(J)** change in HRV averaged over 24 h. Data are means ± standard errors of the mean for three animals. Significant differences relative to baseline (−24–0 h) of each group are shown as ^#^ *p* < 0.05 or ^##^ *p* < 0.01 (two-way analysis of variance, followed by Bonferroni tests). Significant difference between the two groups are shown as *p < 0.05 or **p < 0.01 (two-way ANOVA followed by Bonferroni tests).

During baseline recordings (-24–0 h), the animals’ mean arterial BP was 101.6 ± 3.9 mmHg (systolic BP 123 ± 3.7 mmHg; diastolic BP 90.7 ± 4.9 mmHg; pooled data, *n* = 6), and their HR and HRV were 332.3 ± 33.6 bpm and 0.053 ± 0.013, respectively (pooled data, *n* = 6). Compared with treatment with vehicle, treatment with cisplatin caused a significant reduction in the systolic BP during the 0–24-h (130.04 vs. 111.23 mmHg, *p* < 0.01), 24–48-h (130.53 vs. 107.38 mmHg, *p* < 0.01) and 48–72-h (127.20 vs. 111.17 mmHg, *p* < 0.05) post-treatment periods (Figures 5A,B), as well as a significant reduction in the mean arterial BP during the 0–24-h (106.40 vs. 92.31 mmHg, *p* < 0.05), 24–48-h (105.61 vs. 86.93 mmHg, *p* < 0.01) and 48–72 h (101.99 vs. 89.17 mmHg, *p* < 0.05) post-treatment periods (Figures 5E,F). Moreover, treatment with cisplatin led to a decrease in diastolic BP during the 24–48-h (93.15 vs. 76.70 mmHg, *p* < 0.05) and 48–72-h (89.37 vs. 78.17 mmHg, *p* < 0.05) post-treatment periods (Figures 5C,D), as well as a decrease in HR during the 24–48-h (374.60 vs. 327.46 bpm, *p* < 0.05) and 48–72-h (374.60 vs. 315.78 bpm, *p* < 0.01) post-treatment periods (Figures 5G,H). Neither vehicle nor cisplatin treatment had an effect on HRV throughout the experiments (*p* > 0.05) (Figures 5I,J).

### *In Vivo* Effects of Cisplatin on Respiratory Activity

**FIGURE 6:**
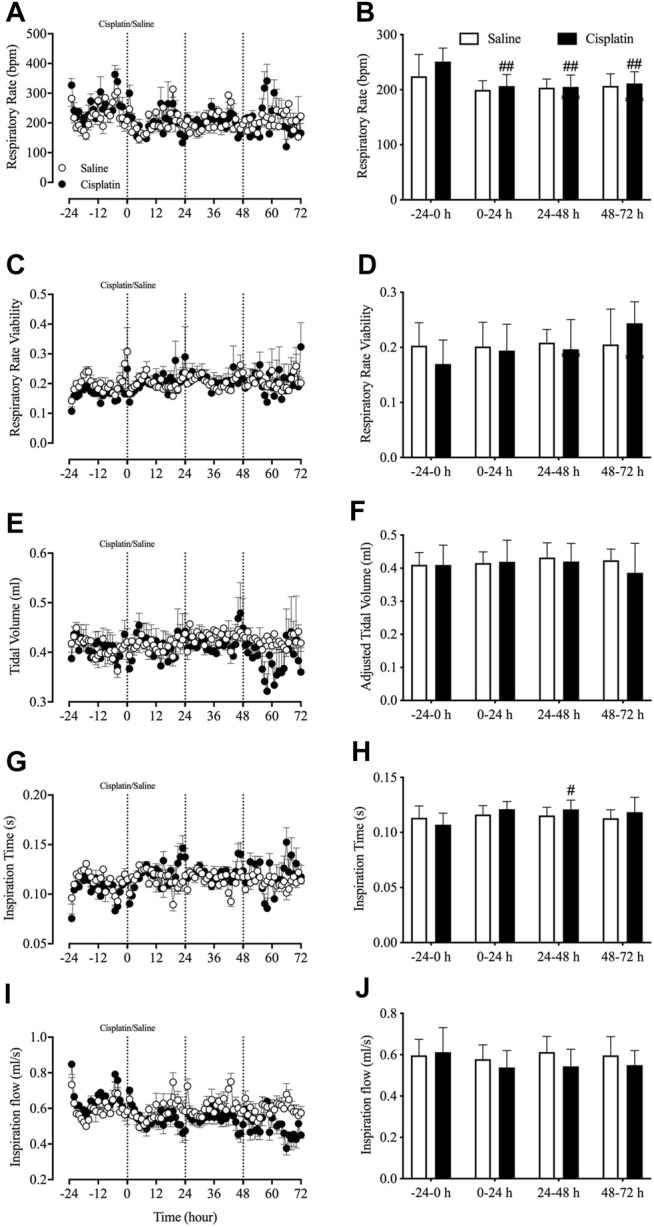
Effect of cisplatin (30 mg/kg, intraperitoneal) on respiratory activity in *Suncus murinus*. **(A)** Respiratory rate in breaths per min (bpm); **(B)** change in respiratory rate averaged over 24 h; **(C)** respiratory rate viability; **(D)** change in respiratory rate viability averaged over 24 h; **(E)** adjusted tidal volume; **(F)** change in adjusted tidal volume averaged over 24 h; **(G)** inspiration time; **(H)** change in inspiration time averaged over 24 h; **(I)** inspiration flow; and **(J)** change in inspiration flow averaged over 24 h. Data are means ± standard errors of the mean for *n* = 7 – 8 animals. Significant differences relative to baseline (−24– 0 h) in each group are shown as ^#^ *p* < 0.05, or ^##^ *p* < 0.01 [two-way analysis of variance (ANOVA) followed by Bonferroni tests]. There was no significant difference between the two groups *p* > 0.05 (two-way ANOVA followed by Bonferroni tests).

Prior to randomisation to different treatment groups, the basal data revealed that the animals’ respiratory rate was 236.9 ± 9.1 bpm, respiratory rate viability was 0.19 ± 0.01, tidal volume was 0.41 ± 0.01 ml, inspiration time was 0.11 ± 0.002 s and inspiration flow was 0.60 ± 0.02 ml/s (pooled data, *n* = 15). There were no significant differences in any of these parameters (i.e., respiratory rate and viability, tidal volume, inspiration time and inspiration flow) between the vehicle control and cisplatin treatment groups during the 24-h baseline period. However, compared with baseline, cisplatin treatment caused a significant reduction in the respiratory rate during the 0–24-h (251.14 vs. 206.56 cpm, *p* < 0.01), 24–48-h (251.14 vs. 205.14 cpm, *p* < 0.01) and 48–72-h (251.14 vs. 211.27 cpm, *p* < 0.01) post-treatment periods (Figures 6A,B). Moreover, cisplatin treatment also induced a significant increase in inspiration time during the 24–48-h post-treatment period (0.107 vs. 0.121 s, *p* < 0.05) (Figures 6G,H). Vehicle treatment had no effect on respiratory activity throughout the entire experiment (*p* > 0.05).

### Analysis of Emetic Data Using Burst Analysis

**FIGURE 7:**
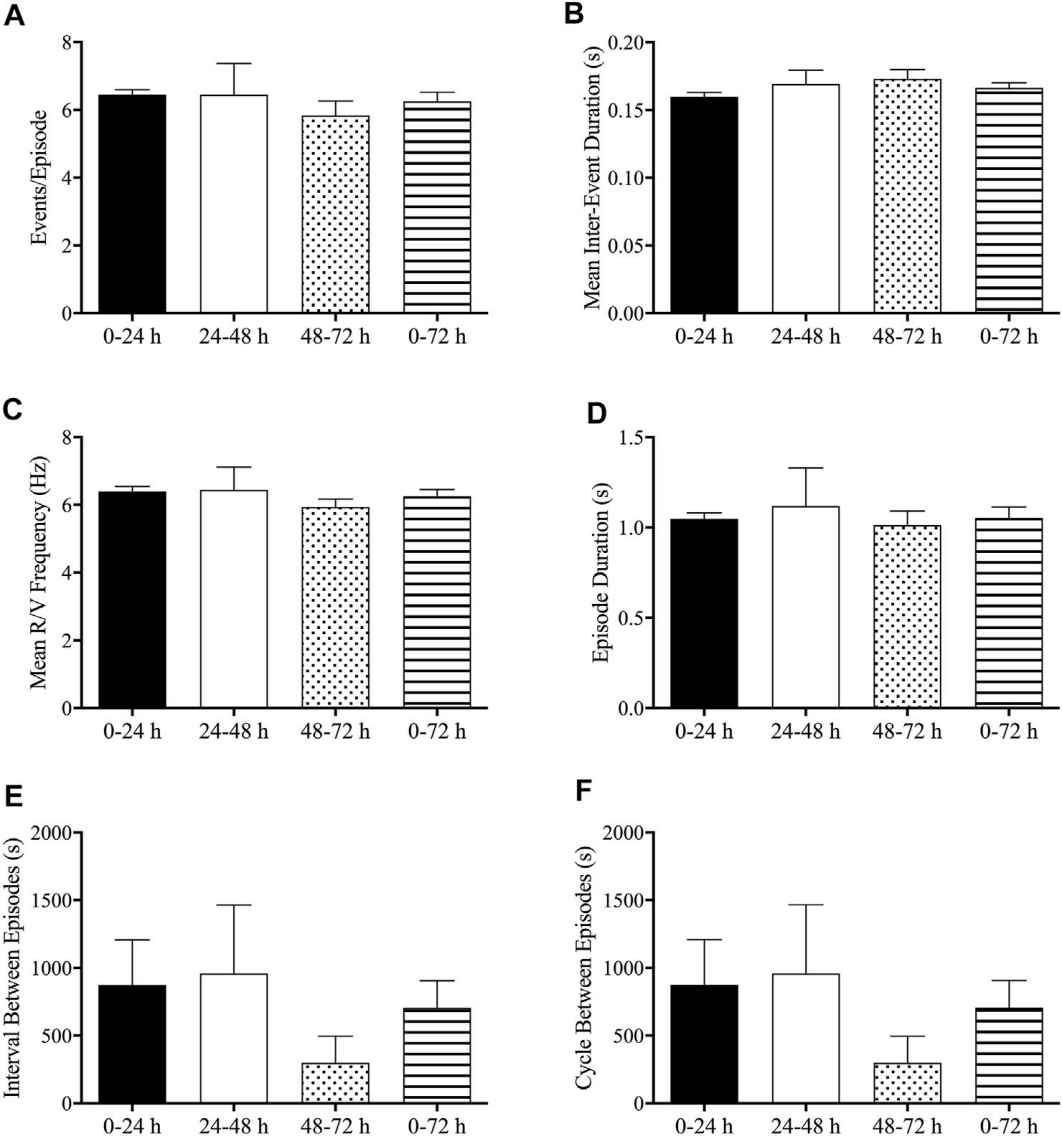
Analysis of emetic data using burst analysis. **(A)** Events per episode/burst; **(B)** mean inter-event duration; **(C)** mean retch/vomit frequency; **(D)** episode durations; **(E)** interval between episodes; **(F)** cycles between episodes. The results are presented means ± standard errors of the mean for *n* = 7 animals.

No significant differences were found in emetic patterns between the events occurring in the 0–24-h, 24–48-h, 48–72-h, and 0–72-h post-treatment periods. Thus, the events/episode, the mean retch + vomit frequency, the emetic episode duration, the intervals between episodes, and the cycles between episodes were fairly consistent (Figures 7A–F).

### Effects of Cisplatin on Morphology and CD45 and c-Kit Expression in the Antrum Region of the Stomach

**FIGURE 8:**
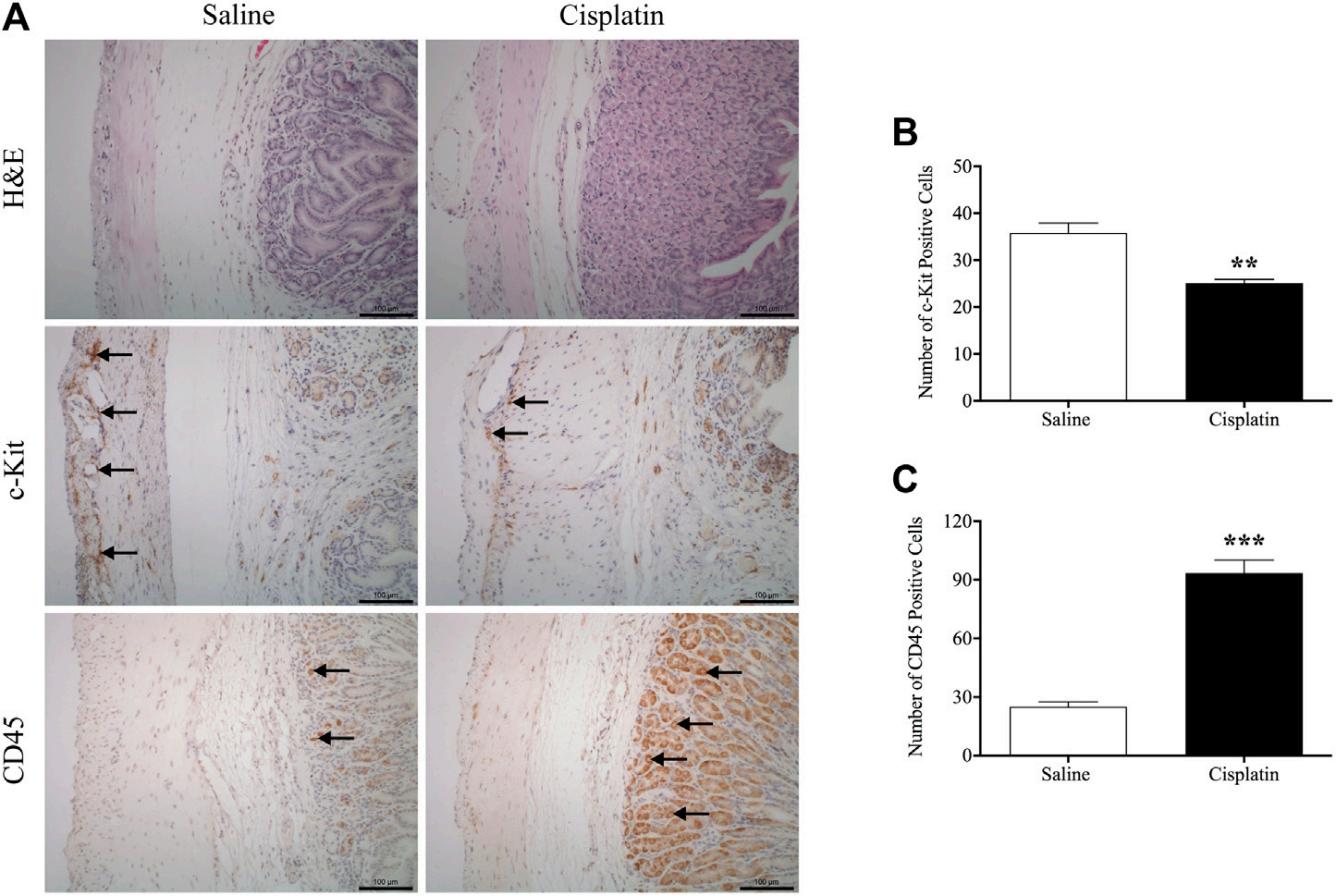
Effects of cisplatin (30 mg/kg, intraperitoneal) on morphology and associated protein expression in the antrum region of the stomach in *Suncus murinus*. **(A)** Representative images of haematoxylin and eosin staining, and immunohistochemistry images of c-kit and CD45; **(B)** changes in the number of c-kit-positive cells; **(C)** changes in the number of CD45-positive cells. Significant differences between cisplatin and saline groups are shown as ^**^ *p* < 0.01 or ^***^ *p* < 0.001 (Student’s *t*-test). The results are presented as means ± standard errors of the mean for *n* = 7 – 8 animals. Positive cells are indicated by black arrows. Scale bar: 100 μm.

We examined the general morphology of the antrum region of the stomach by microscopic analysis of H&E-stained tissue. The gastric antrum (the mucosa, submucosa, circular layer and longitudinal layers) of saline-treated animals appeared normal. Cisplatin treatment did not modify the morphology of the antrum (Figure 8A). However, a suspected gastric parietal hyperplasia was observed in some sections of cisplatin-treated animals. Immunohistochemistry analysis showed that in comparison with vehicle treatment, cisplatin treatment caused a reduction in c-kit-positive cells (25.0 vs. 35.8 per section, *p* < 0.01) (Figure 8B) and a significant increase in CD45-positive cells (93.4 vs. 25.1 per section, *p* < 0.001) (Figure 8C) in the antrum region of the stomach.

## Discussion

The present investigation was the first to use radiotelemetry to collect GMA data, core body temperature, cardiovascular activity data, and respiratory activity data in a single experiment to examine the side effects of cisplatin in *S. murinus*. We also used an MEA technique to characterise the acute *in intro* and *ex vivo* effects of cisplatin on pacemaker activity along the gastrointestinal tract of *S. murinus*.

We found that cisplatin (1–10 μM) had acute (within minutes) region-dependent effects on the pacemaking activity along the gastrointestinal tract of *S. murinus*. The stomach and colon responded to cisplatin treatment in an opposite fashion to the duodenum and ileum, as cisplatin (10 μM) increased the DF in the stomach and colon, but decreased the DF in the duodenum and ileum. Changes in the DF appeared to inversely correlate with changes in the propagating velocity. For example, in the colon, cisplatin (10 μM) significantly increased the DF and simultaneously decreased the propagating velocity. How these changes in electrical pacemaking signals physiologically affect gut motility remains unknown, but the rapid development of regional differences is intriguing. Cisplatin may have been more rapidly concentrated in the duodenum and ileum than in the stomach and colon, because of the difference between the cell types in these areas; if this is the case, this may be part of a mechanism contributing to emesis. We suggest that the cisplatin-induced acute dysrhythmia that we observed *in vitro* may increase over the long term and may contribute to the mechanisms of emesis and changes in gastric function observed in cancer patients treated with cisplatin (Clavel et al., 1987).

It is known that cisplatin affects the functions of several ion channels. For example, cisplatin (0.5 μM) increased currents via N-type voltage-gated Ca^2+^ channels in dorsal root ganglion cells in rats, and caused significant damage to those cells after a 26-day treatment (Leo et al., 2017). No studies have indicated that N-type voltage-gated Ca^2+^ channels play a role in gastrointestinal (GI) slow-wave activities, but inhibitors of N-type voltage-gated Ca^2+^ channels have been used to treat GI dysmotility and chronic visceral pain (McMillan and Srinivasan, 2010; Sadeghi et al., 2018). Cisplatin was also shown to be a non-competitive inhibitor of the mechanosensitive Na^+^/H^+^ exchanger NHE-1 and to block the Cl^–^- and K^+^-mechanosensitive ion channels VSORC and TREK-1 with an ID_50_ of 30 μg/ml (Milosavljevic et al., 2010). However, these ion channels are not known to be expressed in ICCs in the mouse (Lee et al., 2017). Cisplatin (0.5 mM) also induced K^+^ currents in a murine colon carcinoma cell culture, which can be blocked by tetraethylammonium chloride (Sharma et al., 2016). The possible direct effects of cisplatin on ion channels expressed in ICCs require further investigation. Alternatively, the region-dependent effects of cisplatin in our studies may be explained by the differential expression of many ion channels in the jejunum, colon and other regions in the mouse (Lee et al., 2017).

The time to reach peak emesis following cisplatin treatment in *S. murinus* was ∼90 min after i.p. injection (Supplementary Figure S2). At this time point, the DF (measured using an *ex vivo* MEA technique) was generally reduced along the entire GI tract, with a significantly lower DF in the ileum of cisplatin-treated animals compared to saline control-treated animals. The period of waveforms was also significantly reduced in the ileum and colon 90 min after cisplatin treatment. The reductions in the DF in the ileum were similar to those seen in the *ex vivo* study, but there were no changes in the DF in the colon or stomach.

It is not surprising that cisplatin inhibited food and water intake and caused weight loss during the early and delayed phases, as this is highly consistent with data from studies using ferrets (Yamamoto et al., 2004; Lu et al., 2017a). Moreover, the inhibition of food intake and/or anorexia and weight loss caused by cisplatin has been observed in a range of mammals (Liu et al., 2005; Malik et al., 2007). The mechanisms by which cisplatin inhibits food intake and causes weight loss are unknown. However, recent data highlighted that the activation of dorsal vagal complex neurons that project to the lateral parabrachial nucleus (IPBN), and a population of activated IPBN calcitonin gene-related peptide (CGRP) neurons that project to the central nucleus of the amygdala mediate cisplatin-induced malaise and energy-balance dysregulation (Alhadeff et al., 2015). Further studies revealed that excitatory hindbrain-forebrain communication mediated the anorexia and weight loss caused by cisplatin in rats (Alhadeff et al., 2017). Our previous studies in *S. murinus* also demonstrated that cisplatin caused an increase in c-fos expression in the area postrema and the nucleus of the solitary tract, which might also contribute to the inhibition of food intake (Chan et al., 2014). Available evidence suggests that anorexia, as well as “nausea” and emesis induced by cisplatin is associated with increase of growth differentiation factor 15 (GDF15) (Borner et al., 2020). Hsu et al., have shown that GDF15 receptor, derived neurotrophic factor (GDNF) receptor alpha-like (GFRAL) knockout mice are resistant to chemotherapy-induced anorexia and body weight loss (Hsu et al., 2017). Further evidence revealed that GDF15 signals via activation of cholecystokinin neurons in the area postrema and nucleus of the tractus solitarius may contribute to anorexia and body weight loss (Worth et al., 2020).

In the presence of emesis, cisplatin has been shown to cause gastric dysrhythmia in ferrets and dogs (Chey et al., 1988; Percie du Sert et al., 2009; Yu et al., 2009; Lu et al., 2017a). In ferrets, cisplatin caused a transient increase in the DF and a decrease in the DP in the first 8 h after treatment; a significant reduction in the percentage power of normogastria, concomitant with an increase in the percentage power of tachygastria, was also observed (Lu et al., 2017a). Furthermore, there was a decrease in the DP, shown by a 74.5% reduction in the percentage power of bradygastria 12 h after treatment (Lu et al., 2017a). A study in dogs revealed that cisplatin [1.2 mg/kg, intravenous (i.v.)] disrupted gastric and intestinal inter-digestive myoelectric activity for at least 24 h (Chey et al., 1988). Another dog study showed that cisplatin (1.5 mg/kg, i.v.) significantly reduced the time for which the GMA frequency was in the normal range (Yu et al., 2009). However, these two studies did not provide a further analysis of GMA, unlike the studies on ferrets. In the present study in *S. murinus*, we found that cisplatin decreased the DF and increased the percentage power of bradygastria during the acute and delayed phases. The reason for the different effects of cisplatin in GMA in dogs, ferrets and *S. murinus* is unknown. When we looked at a peri-analysis of data around emetic episodes in (Supplementary Figure S4), a shift to tachygastria was more prominent for *Suncus murinus* than seen in in our previous studies in ferrets (Percie du Sert et al., 2009). Species differences, the dose and the route of cisplatin administration, and the analytical methods used may account for the differences observed between studies.

A gastric slow-wave is a summation of voltages that are initiated by ICCs and smooth muscle (Koch and Stern, 2004). However, there is no evidence that cisplatin directly reacts with ICCs or smooth muscle. Our MEA data revealed that 10 μM cisplatin increased the DF in the stomach, whereas 1 and 10 μM cisplatin increased the tachy-rhythm percentage and decreased the normal-rhythm percentage in the stomach. There were differences in the effect of cisplatin between assessments made on tissues excised (‘*ex vivo*’) and assessed on the microelectrode array, and from the radiotelemetric recordings made *in vivo*. A local release of 5-HT and other mediators from enterochromaffin cells in the GI tract may be envisaged to activate/sensitize vagal afferents to modulate neurons in the nucleus tractus solitarius in the brainstem resulting in nausea and vomiting (Sanger and Andrews, 2018). There may be other mediators released into the circulation and/or change in function of efferent nerves impinging on ICCs. However, once tissues are excised, the ICCs would no longer be exposed to circulating factors, or be under the control of efferent nerve modulation from the CNS or other indirect mechanisms. Therefore, this may explain the differences that we observed.

In the present study, hypothermia was observed in *S. murinus* in the 0–24-h and 24–72-h post-cisplatin treatment periods, which has not been previously reported. In contrast, hyperthermia was observed in a previous study of ferrets in the 0–8-h post-cisplatin treatment period, followed by hypothermia in the 24–72-h post-cisplatin treatment period (Lu et al., 2017a). There is also evidence that chronic treatment with cisplatin induced hypothermia in rats (Guindon and Hohmann, 2013). The underlying mechanism of cisplatin-induced hypothermia might be related to the anorexia and lowered energy expenditure caused by cisplatin. Another pro-emetic stimulus, provocative motion, has also been shown to cause hypothermia in laboratory animals (e.g., rats, *S. murinus* and mice) (Ngampramuan et al., 2014; Tu et al., 2017b). However, it is unclear whether hypothermia is an indicator of a nausea-like state in such animals, given the complexity of mechanisms involved in nausea in humans and animals (Andrews and Sanger, 2014; Nalivaiko et al., 2014).

The present study was the first to record BP and HR in conscious *S. murinus*. However, we only used three animals in each vehicle- and cisplatin-treated group, as this enabled us to record BP and HR continuously (as the catheter of the HD-X11 transmitter did not fit well into the *S. murinus* carotid). Nevertheless, our data showed that treatment with cisplatin caused a significant reduction in systolic, diastolic and mean arterial BP in the early (0–24-h) phase and the delayed (24–72-h) phase of emesis in *S. murinus*. Our previous studies in ferrets demonstrated that cisplatin did not change BP in the acute or delayed phase of emesis (Lu et al., 2017a). Furthermore, in the present study, we found that cisplatin reduced HR during the delayed phase of emesis, but had no effect on HRV; in contrast, in ferrets we found that cisplatin decreased HR and HRV only during the acute phase of emesis (Lu et al., 2017a). The reason for these discrepancies in the cardiovascular effects of cisplatin in ferrets and *S. murinus* is unknown. The species difference may be responsible, even though both species have been extensively used for research on emesis. This is supported by our previous finding that the combined ability of ondansetron and dexamethasone to reduce acute and delayed emesis is not seen in *S. murinus*, but is seen in other species (Sam et al., 2003).

Several studies have shown that respiratory function is disturbed during nausea and interrupted during emesis (Horn et al., 2016; Gavgani et al., 2017). Our previous studies in shrews have revealed that provocative motion induces a significant increase in the respiratory rate, concomitant with a reduction in tidal volume (Tu et al., 2017a; Tu et al., 2020). In the present study, we found that cisplatin treatment of *S. murinus* caused a reduction in the animals’ respiratory rate, while other parameters remained unchanged. A study conducted in rats illustrated that lithium chloride, a commonly used and well-characterised pro-emetic compound that robustly induces vomiting in *S. murinus* and ferrets, caused a significant reduction in the respiratory rate (Ngampramuan et al., 2013). The disturbance of respiratory function in these contexts suggest that respiratory function may be associated with a “nausea-like” state and/or emesis in animals.

Lastly, we demonstrated that treatment with cisplatin causes inflammation in the antrum of the stomach in *S. murinus*. This finding is not surprising, because a single 6 mg/kg dose of cisplatin was found to be sufficient to cause mucosal damage in the GI tract of rats (Yamamoto et al., 2013). It was suggested that in addition to 5-HT, inflammatory mediators released in the GI tract may be involved in cisplatin-induced emesis and loss of appetite (Andrews and Rudd, 2015). Interestingly, in the present study, we also found that cisplatin caused a decrease in c-kit expression in the antrum of *S. murinus*, which may indicate a reduction in ICC density. It is not known if this reflects a direct action of cisplatin on ICCs, and we did not examine if cisplatin caused changes in inflammatory markers or ICC density in other regions of the GI tract.

One limitation of the present study was whether observed effects were caused by cisplatin directly or were a consequence of reduction in food intake. Whilst a pair-fed group would be useful in the future to address this possibility, it would be expected to have different consequences. For example, fasting is known to have beneficial effects on blood pressure and heart rate variability (Nicoll and Henein, 2018) and also produces a reduction in the power of slow waves (Lu et al., 2014).

In conclusion, the present study revealed that cisplatin exerts acute region-dependent effects on the pacemaking activity along the gastrointestinal tract of *S. murinus*. Moreover, this is the first study to have simultaneously quantified gastric GMA, respiratory function, body temperature, and cardiovascular (BP, HRV) and behavioural (food and water intake) effects of cisplatin, in addition to its emetic effects, over a 72-h period encompassing acute and delayed emetic phases. The results provide novel insights into events that are likely to occur in patients undergoing cisplatin-based anticancer chemotherapy.

## Data Availability

The original contributions presented in the study are included in the article/Supplementary Material, further inquiries can be directed to the corresponding author.

## Supplementary Material

Supplementary Figure 1 Representative raw trace of pacemaker potentials at baseline (black) and during cisplatin (1 – 10 μM; red or blue) treatment. Red and blue lines represent data showing an increase or decrease in the dominant frequency, respectively.

Supplementary Figure 2 The profile of cisplatin- and vehicle-induced retching + vomiting in *Suncus murinus* during a 72-h observation period. Results are plotted as means ± standard errors of the mean for n = 7 animals.

Supplementary Figure 3 Multifractal detrended fluctuation analysis of gastric myoelectric activity (GMA) in *Suncus murinus*. **(A)** Generalised Hurst exponent h(q) vs. order q, and **(B)** dimension of subset series f(α) vs. singularity strength α for GMA in one animal during four different time-periods: pre-cisplatin treatment (-24 – 0 h) and post-cisplatin treatment (0 – 24 h, 24 – 48 h and 48 – 72 h); **(C)** the width of the multifractal spectrum (Δα) in four different periods (-24 – 0 h, 0 – 24 h, 24 – 48 h and 48 – 72 h) in the vehicle and cisplatin groups are shown, and data are means ± standard errors of the mean for n = 7 – 8 animals. No significant differences were found between the saline and cisplatin groups [one-way analysis of variance (ANOVA) or two-way ANOVA, as appropriate].

Supplementary Figure 4 Dominant frequency **(A)** and repartition of gastric myoelectric activity in the bradygastric **(B)**, normogastric **(C)** and tachygastric **(D)** ranges during baseline, immediately before emetic episodes, during emetic episodes and immediately after emetic episodes induced by cisplatin (30 mg/kg, i.p.) in *Suncus murinus* (see Method 2.7.2, for methodology). Results are shown as means ± standard errors of the mean for n = 7 animals. Significant differences compared to baseline are shown as **p < 0.0 or *p < 0.05 (one-way ANOVA followed by Bonferroni tests).

## Abbreviations

BP, blood pressure; DF, dominant frequency; DP, dominant power; GI, gastrointestinal; GMA, gastric myoelectric activity; HR, heart rate; HRV, heart rate viability; ICCs, interstitial cells of Cajal; MEA, microelectrode array; MFDFA, multifractal detrended fluctuation analysis; RSA, running spectrum analysis.
