# Supplementary figures and images for: Insights Into Acute and Delayed Cisplatin-Induced Emesis From a Microelectrode Array, Radiotelemetry and Whole-Body Plethysmography Study of Suncus murinus (House Musk Shrew)
Source: Front Pharmacol. 2021 Dec 3;12:746053. doi: 10.3389/fphar.2021.746053 (PMC8678571; doi:10.3389/fphar.2021.746053)

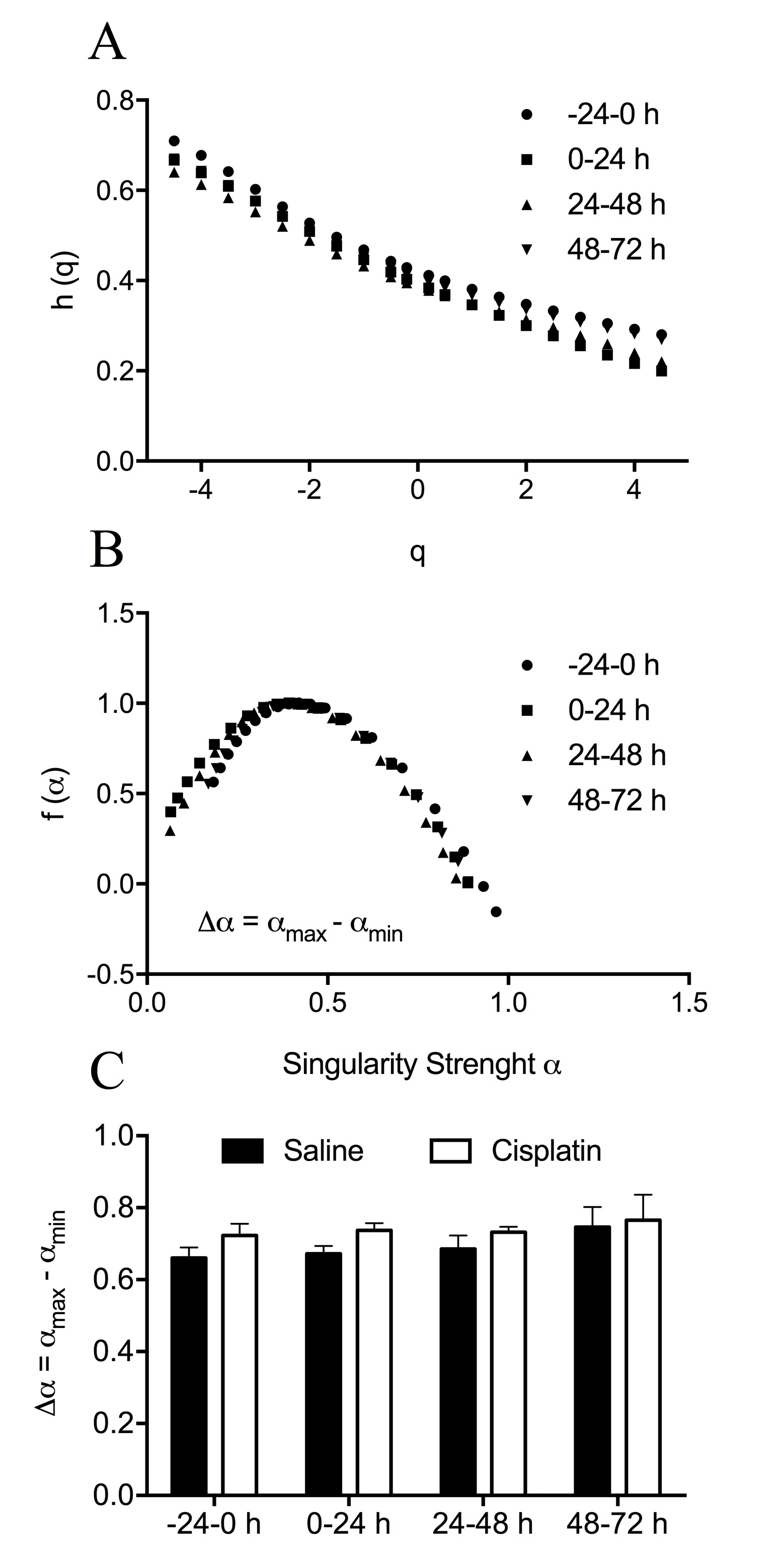

Supplement: Supplementary file 1 [file Image3.JPEG]

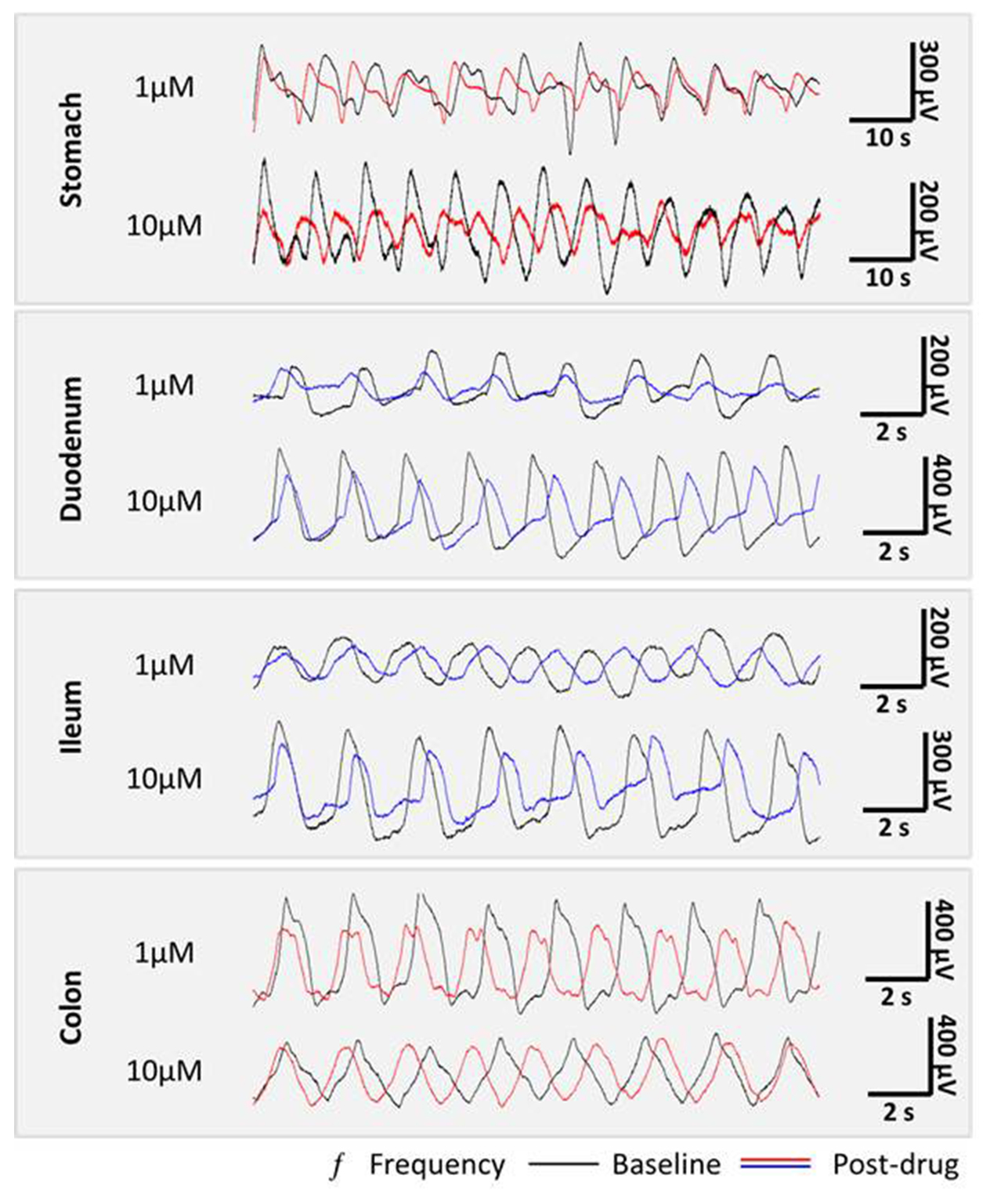

Supplement: Supplementary file 2 [file Image1.JPEG]

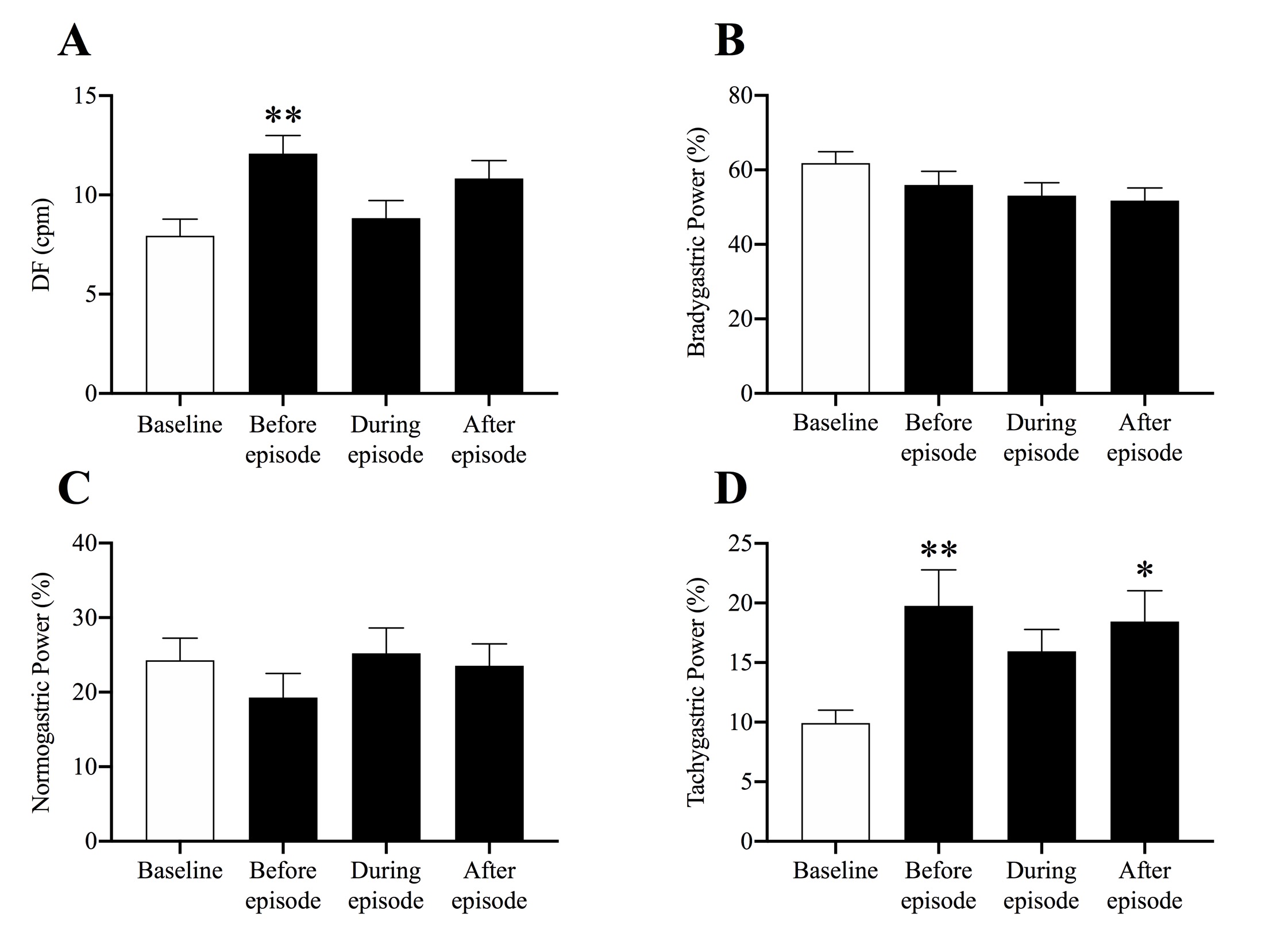

Supplement: Supplementary file 3 [file Image4.JPEG]

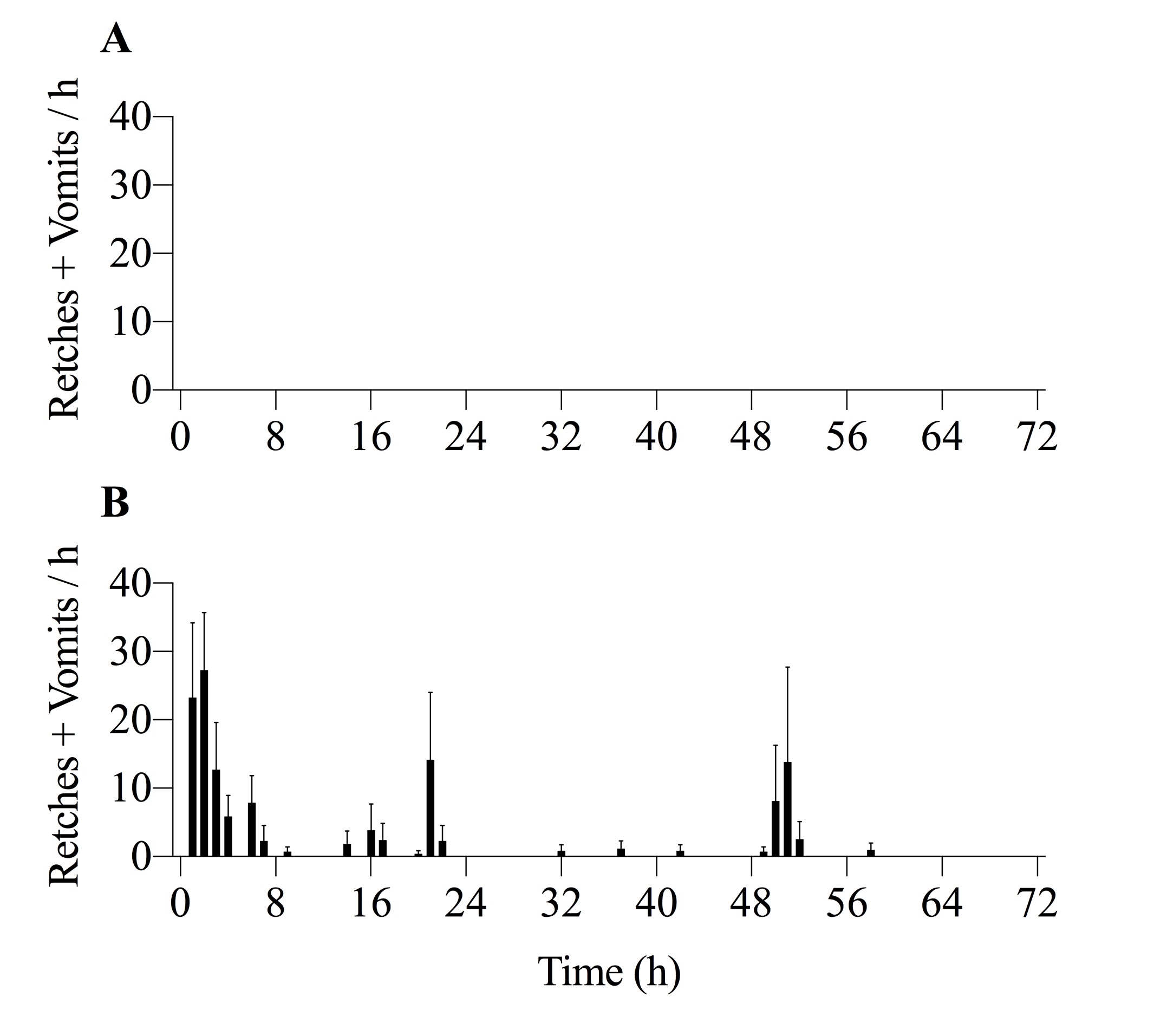

Supplement: Supplementary file 4 [file Image2.JPEG]
