# Supplementary material for: Insights Into Acute and Delayed Cisplatin-Induced Emesis From a Microelectrode Array, Radiotelemetry and Whole-Body Plethysmography Study of Suncus murinus (House Musk Shrew)
Source: Front Pharmacol. 2021 Dec 3;12:746053. doi: 10.3389/fphar.2021.746053 (PMC8678571; doi:10.3389/fphar.2021.746053)
Supplement: Supplementary file 5 [file DataSheet1.docx]

var data%;

Var chan%; 'Channel to process

Var Out%; 'Output channel

var xydata%;

var xymeta%;

var xyhidden%;

var xyspec%;

var FName$;

var Points% := 1; 'how many data points we generate

var Pi := 3.14159265358979323; 'Pi

var e := 2.718281828;

var dataarr1[Points%]; ' sampled data

var dataarr2[Points%]; ' integrated data

var results[20][2]; ' s vs Fq(s)

var svals%[20];

var qvals[20];

var hvals[20];

var tauvals[20];

var alphavals[19];

var Falphavals[19];

setupq();

LogSetUp%();

DoToolbar();

Halt;

'''''''''''''''''''''''''''''''''''''''''''''''''''''''''''''''''''''''''''''

'

funC LogSetUp%()

View(LogHandle());

EditSelectAll();

EditClear();

Window(80,0,100,100);

WindowVisible(1);

return 1;

end;

'''''''''''''''''''''''''''''''''''''''''''''''''''''''''''''''''''''''''''''

'

proc DoToolbar()

ToolbarSet(1,"&Quit",Quit%);

ToolbarSet(3,"&Open File", OpenFile%);

ToolbarSet(4,"&Integrate", Int%);

ToolbarSet(5,"&MFDFA", MFDFA%);

ToolbarEnable(1,1);

ToolbarEnable(3,1);

ToolbarEnable(4,1);

ToolbarEnable(5,1);

Toolbar("", 1023);

end;

'''''''''''''''''''''''''''''''''''''''''''''''''''''''''''''''''''''''''''''

'

func Quit%()

'SampleStop();

FileClose(-1,-1);

return 0;

end;

'''''''''''''''''''''''''''''''''''''''''''''''''''''''''''''''''''''''''''''

'

func OpenFile%()

Var ok%;

Var Start, EndT; 'Analysis range

data% :=FileOpen("",0,3);

'data% :=FileNew(0,1);

if data% < 0 then Message(Error$(data%)); return 1; endif; 'back to toolbar rather than halt

FName$ := FileName$(3);

FrontView(data%);

DlgCreate("Setup"); 'Start new dialog

DlgChan(1,"Channel to process",513);

DlgButton(0,"Cancel");

DlgButton(1,"OK");

ok% := DlgShow(chan%);

Cursorset(2);

CursorLabel(4,1,"Start");

CursorLabel(4,2,"End");

Interact("Postion cursors at start and end of area to analyse", 1023);

CursorRenumber();

Start:=Cursor(1);

EndT:=Cursor(2);

var time := EndT-Start;

Points% := time / BinSize(chan%);

resize dataarr1[Points%];

resize dataarr2[Points%];

Chandata(chan%, dataarr1[], Start, EndT);

setupS(10, 20);

XRange(Start, EndT);

Optimise();

return 1;

end;

'''''''''''''''''''''''''''''''''''''''''''''''''''''''''''''''''''''''''''''

'

func Int%()

ArrConst(dataarr2[],dataarr1[]);

var mean;

ArrSum(dataarr2, mean);

ArrSub(dataarr2, mean);

ArrIntgl(dataarr2);

Out%:=ChanNew(0, 9, 0, BinSize(chan%));

If Out%< 0 then

Message("Could not create channel. Halting");

Halt;

endif

ChanWriteWave(Out%, dataarr2, 0);

Optimise();

return 1;

end;

'''''''''''''''''''''''''''''''''''''''''''''''''''''''''''''''''''''''''''''

'

func MFDFA%()

var q ;

var qi% := 0;

xydata% := Filenew(12,0);

view(xydata%).WindowTitle$("s vs. F_q(s)");

xymeta% := Filenew(12,0);

view(xymeta%).WindowTitle$("q vs. h(q)");

xyhidden% := Filenew(12,0);

var si%;

var ret;

var s%;

var Ns%;

var deg%;

var i%;

var sTime, eTime;

var FSum;

var FqSum;

var F;

var c; 'running error for kahan summation

var y, t; ' also used for kahan summation

for qi% := 0 to 19 do

q := qvals[qi%];

PrintLog(Print$("q = %0.2f\t", q));

yield();

si% := 0;

for si% := 1 to 20 do

s% := svals%[si%-1];

Ns% := Points%/s%;

deg% := 1;

FSum := 0;

FqSum := 0;

F := 0;

c := 0; 'running error for kahan summation

y := 0;

t := 0; ' also used for kahan summation

for i% := 0 to Ns%-1 do

sTime := i%*s%;

sTime *= view(data%).BinSize(chan%);

eTime := min((i%+1)*s%, Points%);

eTime *= view(data%).BinSize(chan%);

view(data%).ChanFit(Out%, 2, deg%); 'Initialise channel fit

view(data%).ChanFit(Out%, 1, sTime, eTime, 0); 'Fit to channel data

F := view(data%).ChanFit(Out%, -4)/s%;

FSum += F;' never used

'what we would like to do is FqSum += pow(F, q/2)

' but this can cause errors due to rounding

' when pow(F, q/2) is small so instead we do kahan summation

' and hope for the best!

if F <> 0

then

y := pow(F, q/2) - c;

t := FqSum + y;

c := (t - FqSum) - y;

FqSum := t;

endif;

next;

' go backwards

for i% := 0 to Ns%-1 do

sTime := max(0,Points%-(i%+1)*s%);

sTime *= view(data%).BinSize(chan%);

eTime := Points%-i%*s%;

eTime *= view(data%).BinSize(chan%);

view(data%).ChanFit(Out%, 2, deg%); 'Initialise channel fit

view(data%).ChanFit(Out%, 1, sTime, eTime, 0); 'Fit to channel data

F := view(data%).ChanFit(Out%, -4)/s%;

FSum += F;' never used

if F <> 0

then

y := pow(F, q/2) - c;

t := FqSum + y;

c := (t - FqSum) - y;

FqSum := t;

endif;

next;

FqSum /= (2.0*Ns%);

ret := pow(FqSum, 1.0/q);

results[si%-1][0] := s%;

results[si%-1][1] := ret;

yield();

next;

' do plots

if qi% <> 0 then view(xydata%).XYSetChan(0) endif;

if qi% <> 0 then view(xyhidden%).XYSetChan(0) endif;

view(xydata%).XYJoin(qi%+1, 1);

view(xydata%).ChanColourSet(qi%+1, 1, Rand(), Rand(), Rand());

view(xydata%).ChanTitle$(qi%+1, Print$("q = %0.2f", q));

for si% := 1 to 20 do

view(xydata%).XYAddData(qi%+1, results[si%-1][0], results[si%-1][1]);

view(xyhidden%).XYAddData(qi%+1, log(results[si%-1][0]), log(results[si%-1][1]));

next;

' calc h(q)

var k := 0;

view(xyhidden%).ChanFit(qi%+1, 2, 1);

view(xyhidden%).ChanFitShow(qi%+1, 1); 'Set up fit display

view(xyhidden%).ChanFit(qi%+1, 1, log(results[0][0])-1, log(results[19][0])+1, log(results[0][0])-1); 'Fit to channel data

k := view(xyhidden%).ChanFitCoef(qi%+1, 1);

hvals[qi%] := k;

PrintLog(Print$("h(q) = %0.4f\n", k));

next;

' Now calculate tau values

var j%;

for j% := 0 to 19 do

tauvals[j%] := (hvals[j%] * qvals[j%]) - 1;

next;

' Now calculate the hbar values

for j% := 0 to 18 do

alphavals[j%] := (tauvals[j%]-tauvals[j%+1])/(qvals[j%]-qvals[j%+1]);

next;

' Now calculate the D values

for j% := 0 to 18 do

Falphavals[j%] := qvals[j%] * alphavals[j%] - tauvals[j%];

next;

' Now plot multi-fractal spectrum

xyspec% := Filenew(12,0);

view(xyspec%).WindowTitle$("multi-fractal spectrum");

view(xyspec%).XYAddData(1, alphavals[], Falphavals[]);

view(xymeta%).XYJoin(1, 1);

view(xymeta%).XYAddData(1, qvals[], hvals[]);

view(xymeta%).ChanTitle$(1, "q vs. h(q)");

view(xydata%).WindowVisible(1);

view(xymeta%).WindowVisible(1);

view(xyspec%).WindowVisible(1);

view(data%).ChanDelete(Out%);

view(xydata%).XAxisStyle(1, 0, 0);

view(xydata%).XAxisAttrib(1);

view(xydata%).YAxisStyle(-1, 0, 0);

view(xydata%).YAxisAttrib(-1, 3);

view(xydata%).Optimise(-2);

view(xymeta%).Optimise(-2);

PrintLog("S values - \n");

for si% := 0 to 19 do

PrintLog("%d", svals%[si%]);

if si% <> 19 then

PrintLog(", ");

else

PrintLog("\n");

endif

next;

end;

'''''''''''''''''''''''''''''''''''''''''''''''''''''''''''''''''''''''''''''

'

func setupS(minpts%, minints%)

var MinS% := minpts%;

var MaxS% := Points%/minints%;

var t := Ln(1.0*MaxS%/MinS%)/19;

var rat := exp(t);

var i%;

for i% := 0 to 19 do

svals%[i%] := MinS%*pow(rat, i%);

next;

end;

'''''''''''''''''''''''''''''''''''''''''''''''''''''''''''''''''''''''''''''

'

func setupq()

qvals[0] := -4.5;qvals[1] := -4;qvals[2] := -3.5;qvals[3] := -3;qvals[4] := -2.5;

qvals[5] := -2;qvals[6] := -1.5;qvals[7] := -1;qvals[8] := -0.5;qvals[9] := -0.2;

qvals[19] := 4.5;qvals[18] := 4;qvals[17] := 3.5;qvals[16] := 3;qvals[15] := 2.5;

qvals[14] := 2;qvals[13] := 1.5;qvals[12] := 1;qvals[11] := 0.5;qvals[10] := 0.2;

end;
